# Anyon superconductivity from topological criticality in a Hofstadter–Hubbard model

**DOI:** 10.1073/pnas.2426680122

**Published:** 2025-08-12

**Authors:** Stefan Divic, Valentin Crépel, Tomohiro Soejima, Xue-Yang Song, Andrew J. Millis, Michael P. Zaletel, Ashvin Vishwanath

**Affiliations:** ^a^Department of Physics, University of California, Berkeley, CA 94720; ^b^Center for Computational Quantum Physics, Flatiron Institute, New York, NY 10010; ^c^Department of Physics, Harvard University, Cambridge, MA 02138; ^d^Department of Physics, Hong Kong University of Science and Technology, Clear Water Bay, Hong Kong, China; ^e^Department of Physics, Columbia University, New York, NY 10027; ^f^Materials Sciences Division, Lawrence Berkeley National Laboratory, Berkeley, CA 94720

**Keywords:** superconductivity, anyons, Hubbard model, spin liquids, moiré

## Abstract

We argue that the combination of strong repulsive interactions and high magnetic fields can generate electron pairing and superconductivity. Inspired by the large lattice constants of moiré materials, which make large flux per unit cell accessible at laboratory fields, we study the triangular lattice Hofstadter–Hubbard model at one-quarter flux quantum per plaquette, where previous literature has argued that a chiral spin liquid separates a weak-coupling integer quantum Hall phase and a strong-coupling topologically trivial antiferromagnetic insulator at a density of one electron per site. We argue that topological superconductivity emerges upon doping in the vicinity of the integer quantum Hall to chiral spin liquid transition. We employ exact diagonalization and density matrix renormalization group methods to examine this theoretical scenario and find that electronic pairing indeed occurs on both sides of criticality over a remarkably broad range of interaction strengths. On the chiral spin liquid side, our results provide a concrete model realization of the long-hypothesized mechanism of anyon superconductivity. Our study thus establishes a beyond-Bardeen-Cooper-Schrieffer route to electron pairing in a well-controlled limit, relying crucially on the interplay between electron correlations and band topology.

## Significance

The identification of novel mechanisms for superconductivity is a longstanding goal in physics. We propose that superconductivity emerges naturally near the phase transition between two topologically distinct insulators in a suitable magnetic field. Unlike other mechanisms driven by quantum criticality, our approach enables the numerically controlled demonstration of electron pairing, a crucial precursor to superconductivity. We demonstrate these ideas in a moiré-inspired lattice model of interacting electrons in a magnetic field, providing a concrete realization of the anyon superconductivity mechanism theorized decades ago in the context of the high-*T*_*c*_ cuprates. This work opens pathways for generating superconductivity in the vicinity of topological criticality.

The search for electron pairing mechanisms that go beyond the well-established BCS/“pairing glue” paradigm, exemplified by the electron–phonon mechanism (1–3), has a long history. A popular theoretical route has been to consider situations where charge is associated with topological excitations (4–15). The best known of these proposals are the resonating valence bond (9, 16–18) and anyon superconductivity scenarios (19–28), where doping a quantum spin liquid with fractionalized charge excitations leads to superconductivity. In the anyon superconductivity approach proposed soon after the discovery of high-$T_{c}$ superconductivity in the layered cuprates, the starting point is the chiral spin liquid (CSL) phase introduced by Kalmeyer and Laughlin (29). Although interest in the anyon superconductivity mechanism in the context of high-$T_{c}$ cuprates has waned, it remains a remarkable theoretical example of superconductivity emerging from a chiral insulator.

Spin liquids have proven elusive, and even where they are proposed to appear, superconductivity need not arise upon doping. The triangular lattice Hubbard model with time reversal symmetry has been reported to host a CSL ground state at intermediate coupling (30–33). However, various magnetic orders and other spin liquid phases are so close in energy to the CSL (34–37) that its existence in this model, and in related time reversal-symmetric extended Heisenberg models (36–40), is not definitively established (41, 42). Further, the existence of superconductivity at small hole doping in the intermediate-coupling regime (43, 44) relevant to the putative CSL has not been clearly demonstrated. Indeed, a density matrix renormalization group (DMRG) analysis reports a metal rather than a superconductor (45). In addition, there is no spin liquid in the weak-coupling regime where superconductivity has also been proposed (46–48).

In this work, we show that superconductivity emerges naturally in the vicinity of topological criticality arising from a continuous transition between two topologically distinct insulators, with time reversal symmetry explicitly broken by a magnetic field. The crucial point is that a change in topology requires closing and reopening a gap at the transition, in this case, a charge gap associated with a bosonic charge-2e mode. Near the topological critical point, these bosonic modes are the lowest-energy local charge excitations, while unpaired electronic states appear only at higher energies. Modest doping then introduces paired carriers, which can superconduct.

**Fig. 1.**
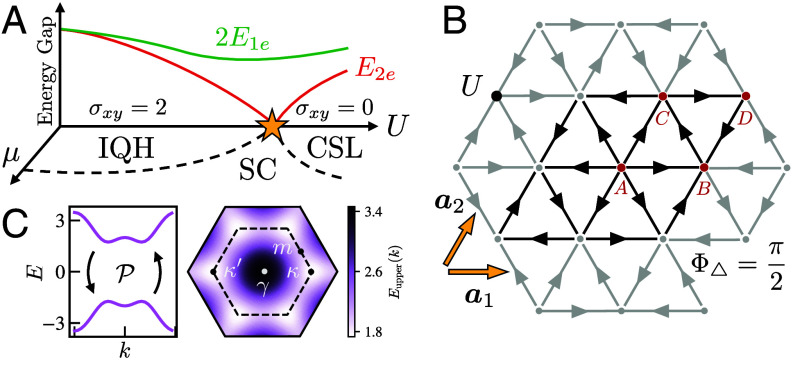
(*A*) Schematic depiction of the predicted energy gap to 2e excitations (red) and twice the energy of 1e excitations (green) at half filling of the Hofstadter–Hubbard model with $\Phi_{▵}=\pi /2$ flux per triangle as a function of interaction strength *U* in the 2D limit. Proposed phase diagram in the plane of *U* and chemical potential *μ*. The star indicates a topological quantum phase transition from an integer quantum Hall (IQH) insulator to chiral spin liquid (CSL) at half filling. The dashed lines show the chemical potential required to add carriers and produce a chiral superconductor (SC) with topologically protected edge modes. (*B*) Finite region of the two-dimensional model with $\Phi_{▵}=\pi /2$ flux per triangle, repulsive on-site interactions *U*, and Bravais vectors $a_{1,2}$ indicated. The hoppings may be chosen to be $C_{6}$-invariant and imaginary (each arrow indicates an amplitude −it), with a four-site unit cell (sublattices *A*-*D* in red). (*C*) *Left*: band structure at $\Phi_{▵}=\pi /2$ consisting of two bands related by particle–hole symmetry P. *Right*: two-dimensional color map of energy as a function of momentum for the upper energy band over the Brillouin zone of the 2×2 unit cell (note the bands are two-fold degenerate at every momentum).

We illustrate the potential of this idea through explicit calculations in a microscopic Hofstadter–Hubbard model argued to host an integer quantum Hall (IQH) phase at weak coupling with charge and spin edge modes, and a CSL phase with only a spin edge mode, arising upon increasing electron correlations via repulsive Hubbard interactions (49, 50). The CSL phase is also numerically observed in extended Heisenberg models with a chiral spin interaction (34–36) arising from the orbital magnetic flux through each plaquette (51, 52). The IQH and CSL insulators appear at a density of one electron per site, henceforth referred to as “half filling,” with superconductivity emerging upon doping electrons or holes. In Fig. 1*A*, we present a schematic phase diagram in the plane of chemical potential *μ* and Hubbard *U*.

Our study goes beyond previous proposals for superconductivity in the Hofstadter flux regime, which considered only attractive (53–59) and perturbatively weak repulsive interactions (60). The investigation of the present strongly repulsive model is motivated by its potential realization in transition metal dichalcogenide (TMD) moiré materials (49, 61), whose advent has opened new avenues for realizing exotic quantum phases and enabled the controlled variation of their doping (62–64). The large unit cells of these materials make the Hofstadter flux regime experimentally accessible, while the Zeeman coupling can be quenched (49).

We present general arguments supported by a parton treatment and effective field theory that reveal low-energy Cooper pairs near the putative IQH-CSL critical point. Furthermore, we contend that doping in its vicinity leads to superconductivity. On the CSL side, the superconductor is shown to arise via the anyon superconductivity mechanism. Pairing is then examined numerically with exact diagonalization (ED) and DMRG, which reveal that charge-2e excitations are indeed the lowest-energy local charge excitations, per unit charge, over a broad range of parameters, a compelling precursor to superconductivity upon doping. Strikingly, the pairing extends beyond the CSL, well into the IQH side of the phase diagram where anyons cannot be invoked, consistent with the softening of the 2e gap by topological criticality.

Importantly, this mechanism does not rely on strict quantum criticality. Superconductivity emerges in a noninfinitesimal neighborhood of the topological critical point and will therefore persist even if the transition is weakly first-order or one of the phases is proximal but avoided. Its precursor, electron pairing, appears even in the finite systems accessible in our numerics. Altogether, our results suggest that superconductivity can arise in a regime with both strong repulsive interactions and broken time reversal symmetry, a combination that normally disfavors superconductivity.

The remainder of the manuscript is organized as follows. In Section 1, we introduce the Hofstadter–Hubbard model as well as its continuous and magnetic space group symmetries. In Section 2, we provide a parton description of the putative topological critical point and the mechanism for obtaining superconductivity upon doping each side of the transition. In Section 3, we provide numerical evidence for electron pairing using both ED and DMRG. Section 4 is a summary and conclusion suggesting avenues for future research. *SI Appendix* provides details of calculations described in the main text and supporting data.

## 1. Model and Symmetries

The Hofstadter–Hubbard model (Fig. 1*B*), with hoppings $t_{\mathrm{ij}}$ from site *j* to *i* and on-site interaction $H_{I}$, is given by $H_{\text{HH}}=-\sum_{\mathrm{ij}}t_{\mathrm{ij}}c_{i}^{†}c_{j}+H_{I}$. The hopping amplitudes $t_{\mathrm{ij}}$ are complex numbers with phases encoding the orbital magnetic flux (65). In this work, we specialize to the triangular lattice with $\Phi_{▵}=\pi /2$ flux per triangle (49, 50) for which the hopping amplitudes can be chosen to be purely imaginary, so that the single-particle term in the Hamiltonian may be written as

[1] $$\begin{array}{c} H_{0}=i\sum_{\left\langle ij\right\rangle }\tau_{\mathrm{ij}}\left(c_{i\sigma }^{†}c_{j\sigma }-c_{j\sigma }^{†}c_{i\sigma }\right). \end{array}$$

The arrows on bonds in Fig. 1*B* indicate our sign convention that the hopping from site *j* to *i* along an arrow has $\tau_{\mathrm{ij}}=-t<0$, while hopping opposite an arrow has the opposite sign. We report energies in units of *t*.

The symmetries of $H_{0}$ are most clearly exposed by mapping to Majorana fermions, $c_{j↑}=\frac{1}{2}\left(\chi_{1}^{j}+i\chi_{2}^{j}\right)$ and $c_{j↓}=\frac{1}{2}\left(\chi_{3}^{j}+i\chi_{4}^{j}\right)$, where $\left\{\chi_{a}^{i},\;\chi_{b}^{j}\right\}=2\delta_{\mathrm{ab}}\delta_{\mathrm{ij}}$ so that $H_{0}$ takes the form (66):

[2] $$\begin{array}{c} H_{0}=\frac{i}{2}\sum_{1\leq a\leq 4}\sum_{\left\langle ij\right\rangle }\tau_{\mathrm{ij}}\chi_{a}^{i}\chi_{a}^{j}, \end{array}$$

which is seen to have an O(4) symmetry, corresponding to proper/improper rotations of the Majorana four-component vector. The Hubbard interaction

[3] $$\begin{array}{c} H_{I}=U\sum_{i}\left(n_{i,↑}-1/2\right)\left(n_{i,↓}-1/2\right) \end{array}$$

can be written in the Majorana representation as $H_{I}=-\left(U/4\right)\sum_{i}\chi_{1}^{i}\chi_{2}^{i}\chi_{3}^{i}\chi_{4}^{i}$. Note that the interaction breaks the O(4) symmetry of the hopping model down to SO(4). The operators in the determinant −1 sector, corresponding to improper rotations, are related to particle–hole transformations that flip the sign of *U* (e.g., conjugating only the spin-up electrons) and are therefore not symmetries of the interacting theory. The particle–hole operation which preserves the sign of *U* is a symmetry, identified in ref. 50.

Contained in SO(4) are the spin ${\text{SU(2)}}_{s}$ and pseudospin ${\text{SU(2)}}_{c}$ symmetry subgroups identified by Yang and Zhang (67–69), and by Affleck (66). While the pseudospin symmetry is typically associated with bipartite lattice Hubbard models (70), we highlight that it is more generally present whenever there exists a gauge in which the hoppings are purely imaginary (71) and that ${\text{SU(2)}}_{c}$ reduces to the usual charge ${\text{U(1)}}_{c}$ for generic long-range interactions. The ${\text{SU(2)}}_{c}$ pseudospin symmetry will play an important role in our study of the topological phase transition in Section 2.1.

The model is additionally symmetric under operations in the magnetic space group generated by the rotation $C_{6}$ and translation $T_{1}$ along the nearest-neighbor direction $a_{1}$ (Fig. 1*B*). Their explicit form, which we provide in *SI Appendix*, section 1B, depends on the choice of gauge for the electron operators. However, they satisfy certain gauge-invariant generating relations which will prove important for interpreting the pairing symmetry in Section 3.3. Because the magnetic space group is a U${\left(1\right)}_{c}$ extension (72), the overall U${\left(1\right)}_{c}$ phase of each spatial symmetry is ambiguous. We make the following convenient choices: We fix ${\left(C_{6}\right)}^{6}=1$, choose $T_{1}$ to satisfy $C_{2}T_{1}C_{2}^{†}=T_{1}^{†}$ which constrains it modulo a sign, and define rotated nearest-neighbor translations by $T_{j+1}=C_{6}^{j}T_{1}C_{6}^{-j}$. Moreover, due to the π/2 flux per triangle, one may verify the following gauge-invariant relations:

[4] $$\begin{array}{cc} T_{2}T_{1} & ={\left(-1\right)}^{N_{F}}T_{1}T_{2},\;T_{5}T_{3}T_{1}={\left(\pm i\right)}^{N_{F}}, \end{array}$$

where $N_{F}$ is the fermion number. The sign ambiguity in the second relation arises because our conventions do not fix the sign of $T_{1}$; we remedy this by choosing +i. When acting on charge-2e pairs, with $N_{F}=2$, the first relation is trivial but the second is not. This is the origin of the unorthodox pairing symmetry we uncover in Section 3.3.

## 2. Parton Theory

### 2.1. Topological Criticality at Half Filling.

We introduce a parton construction that captures the effects of on-site *U*, in effect performing a Gutzwiller projection on the IQH wavefunction to obtain the CSL (73, 74). This formalism reproduces the phase diagram of the half-filled model obtained numerically in refs. 49 and 50 and allows us to study the effects of doping. While it is possible to proceed by retaining the ${\text{SU(2)}}_{c}$ pseudospin symmetry (70), we relegate this treatment to *SI Appendix*, section 6 and, for ease of presentation, derive here a slave-rotor theory retaining only ${\text{U(1)}}_{c}$ along with ${\text{SU(2)}}_{s}$. We begin by introducing a U(1) rotor variable $e^{i\theta }$ and its conjugate integer-valued “angular momentum” *L* at each site (75). We write the electron operator as $c_{i,\sigma }^{†}=f_{i,\sigma }^{†}\;e^{i\theta_{i}}$, where *f* is a fermionic “spinon” carrying the electronic spin. The redundancy introduced by the rotors is alleviated by imposing the following constraint at each site:

[5] $$\begin{array}{c} L_{i}=f_{i,↑}^{†}f_{i,↑}+f_{i,↓}^{†}f_{i,↓}-1. \end{array}$$

Note that a singly filled site is represented by $L_{i}=0$, while the doublon/empty sites correspond to $L_{i}=\pm 1$. The Hubbard model can then be rewritten as (75):

[6] $$\begin{array}{c} H=-\sum_{\langle ij\rangle ,\sigma }t_{\mathrm{ij}}e^{i(\theta_{i}-\theta_{j})}f_{i,\sigma }^{†}f_{j,\sigma }+\text{h.c.}+\frac{U}{2}\sum_{i}L_{i}^{2}. \end{array}$$

To make progress, we adopt a mean field approach, replacing operator bilinears by their mean field values:

[7] $$\begin{array}{c} \begin{array}{cc} H_{\text{MF}} & =H_{f}+H_{\theta }\\ H_{f} & =-\sum_{\langle ij\rangle ,\sigma }{\tilde{t}}_{\mathrm{ij}}f_{i,\sigma }^{†}f_{j,\sigma }+\text{h.c.}\\ H_{\theta } & =-\sum_{\langle ij\rangle ,\sigma }Q_{\mathrm{ij}}e^{i(\theta_{i}-\theta_{j})}+\text{h.c.}+\frac{U}{2}\sum_{i}L_{i}^{2}, \end{array} \end{array}$$

where $Q_{\mathrm{ij}}=t_{\mathrm{ij}}\left\langle f_{i,\sigma }^{†}f_{j,\sigma }\right\rangle$ and ${\tilde{t}}_{\mathrm{ij}}=t_{\mathrm{ij}}\left\langle e^{i(\theta_{i}-\theta_{j})}\right\rangle$. Let us summarize the main results, leaving the detailed analysis of this mean field theory to *SI Appendix*, section 2. Consistency with the numerical results of ref. 50, namely a spin gap throughout the transition, requires that the spinons *f* be gapped. The most natural possibility, consistent with the projective construction of the CSL in the Mott limit (71), is that the spinons see $\Phi_{▵}=\pi /2$ net flux per plaquette, like the microscopic electrons, and enter a spin-singlet Chern insulator with total Chern number C=2. Since the rotor bosons then see no net flux,^*^$H_{\theta }$ displays a superfluid-Mott transition on increasing *U*, corresponding to the electronic IQH-CSL transition. A mean field treatment of $H_{\theta }$ locates this critical point (*SI Appendix*, section 2) at $U^{*}/t\approx 9.6$, close to the transition point $U^{*}/t\approx 12$ estimated by iDMRG (49, 50).

Using Eq. **7**, we show that the universal response properties of the spinon-rotor mean field solutions are consistent with that of the IQH and CSL. We introduce a gauge field on bonds to account for gauge fluctuations about the mean field solution (78), giving $Q_{\mathrm{ij}}\to Qe^{-ia_{\mathrm{ij}}}$ and ${\tilde{t}}_{\mathrm{ij}}\to {\tilde{t}}_{\mathrm{ij}}e^{+ia_{\mathrm{ij}}}$. Further, we introduce a gauge field *A* ($A_{s}$) coupled minimally to the rotors (spinons) to probe the charge (spin) response. Restricting to energies below the spin gap, we may integrate out the spinons; the unit Chern number for each spin species yields the following Chern–Simons terms (79–81):

[8] $$\begin{array}{c} L_{\text{CSL}}=\frac{2}{4\pi }\left(a\wedge da+A_{s}\wedge dA_{s}\right). \end{array}$$

We employ the notation $a\wedge da=\epsilon_{\mu \nu \lambda }a^{\mu }\partial^{\nu }a^{\lambda }$, where summation over Greek spacetime indices is implicit and *ϵ* is the three-dimensional antisymmetric tensor.

We now turn to the rotor charge excitations. Since we are interested here in long wavelength properties, it is convenient to pass to the continuum limit and utilize a coarse-grained “soft-spin” description (82), replacing $\psi ∼e^{i\theta }$ and introducing a potential $V\left(\psi \right)=m^{2}{\left|\psi \right|}^{2}+\lambda {\left|\psi \right|}^{4}$. Note that there is only a single bosonic field *ψ*, corresponding to a single minimum in the rotor dispersion, a consequence of the vanishing average flux experienced by the rotors. Tuning the sign of $m^{2}$ from positive to negative induces condensation of *ψ*, as we describe below. The resulting effective field theory is:^†^

[9] $$\begin{array}{c} L=|D_{A-a}{\psi |}^{2}-V\left(\psi \right)+\frac{2}{4\pi }a\wedge d\;a+\frac{2}{4\pi }A_{s}\wedge d\;A_{s}, \end{array}$$

where $D_{b}^{\mu }=\partial^{\mu }-ib^{\mu }$, and we have taken advantage of field rescaling to bring it into this form. The time component $a_{0}$ is introduced to implement the on-site constraint Eq. **5**. We now consider the two phases and their transition.

#### 2.1.1. Phase I.

$m^{2}<0,\;\left\langle \psi \right\rangle \neq 0$. In the small-*U* limit we expect the rotor variables to “condense,” which introduces a Higgs mass term for a−A. Integrating over *a* yields the response theory $L_{\text{I}}\left[A,A_{s}\right]=\frac{2}{4\pi }A\wedge dA+\frac{2}{4\pi }A_{s}\wedge dA_{s}$. Thus we have an insulator without intrinsic topological order, with precisely the charge and spin quantum Hall conductance of the spin-singlet integer quantum Hall state, namely $\sigma_{\mathrm{xy}}=2\cdot e^{2}/h$ and $\sigma_{\mathrm{xy}}^{s}=2\cdot \hbar /8\pi$. The latter describes the quantized response of spin to a Zeeman gradient, termed the spin quantum Hall effect (85), and is distinct from the quantum spin Hall effect (86, 87).

#### 2.1.2. Phase II.

$m^{2}>0,\;\left\langle \psi \right\rangle =0$. In the larger-*U* regime, the rotor condensate disappears and the *ψ* field is gapped. The low-energy effective action in this phase is then $L_{\text{II}}\left[a,A,A_{s}\right]=\frac{2}{4\pi }a\wedge da+\frac{2}{4\pi }A_{s}\wedge dA_{s}=$$L_{\text{CSL}}$. While the quantized spin response is the same as in Phase I (guaranteed by the spin gap being maintained throughout), the charge Hall conductivity vanishes. Semion topological order now arises from the dynamical U${\left(1\right)}_{2}$ Chern–Simons term. These are the characteristic properties of the CSL phase (71). Moreover, the change of $\sigma_{\mathrm{xy}}$ across the IQH-CSL transition implies that the charge gap must close if the transition is continuous.

#### 2.1.3. Critical point.

In the mean field approximation, the critical point corresponds to setting $m^{2}=0$ in Eq. **9**, so that the *ψ* field becomes gapless. Intuition for this field is gained by recognizing that the fractionalized spin-1/2 semion excitation in the CSL phase combines with the electron to give a semionic charge-*e* spin-0 excitation, represented by the nonlocal *ψ* field. At mean field level, the microscopic particle–hole symmetry ensures that the IQH-CSL transition is continuous and belongs to the 3D XY universality class (82).

To characterize the critical point beyond the mean field approximation, and assess whether the transition remains continuous, we consider gauge field fluctuations and the dynamical Chern–Simons term in Eq. **9**. This full theory cannot be solved exactly, and the Chern–Simons term introduces a sign problem that would hamper large-scale quantum Monte Carlo simulations (88). Alternatively, one can study the microscopic Hamiltonian of Section 1 using tensor network methods. Indeed, the cylinder DMRG studies of refs. 49 and 50 provide direct evidence that the transition is continuous and that electron excitations are gapped across the transition. This points to spin-singlet Cooper pairs being the cheapest local charge excitations near the critical point, which we demonstrate numerically in Section 3.

In addition, we can study deformations of the parton critical theory that can be characterized analytically and are known to exhibit a continuous transition. Specifically, consider Eq. **9** but with Chern–Simons term $\frac{N}{4\pi }a\wedge da$, while retaining the single bosonic scalar field *ψ*. While we are interested in the case N=2, observe that N=1 and N=0 both correspond to continuous transitions: Under fermion-boson duality (89), the former describes the free-fermion Dirac critical point separating two insulators with Chern numbers C=0→1, while the latter describes the 2+1D superconductor-insulator transition (90, 91). Furthermore, in the N=∞ limit, the theory reduces to the 3D XY transition since gauge field fluctuations are suppressed. The existence of a continuous transition in the N=0,1,∞ deformed theories suggests that the N=2 transition can also be continuous.

We shed further light on the IQH-CSL transition by mapping it to an equivalent, better-studied bosonic transition between a trivial insulator and ν=−1/2 Laughlin state of charge-2e Cooper pairs (92–95). The equivalence can be seen by “stacking” an invertible ν=−2 IQH phase above Phases I and II (77). This trivializes both the spin and charge response of the IQH and maps the CSL to a phase with Hall conductance $\sigma_{\mathrm{xy}}=-\frac{1}{2}\frac{{\left(2e\right)}^{2}}{h}$ but no spin response, namely a Laughlin liquid of Cooper pairs. The transition can thus be viewed as a plateau transition of Cooper pairs, rationalizing there being gapless Cooper pairs at the critical point.

This bosonic transition is argued to be continuous, with critical exponents and universal conductivity computed perturbatively, in several works (92, 93, 96). In contrast, ref. 97 proposes a first-order transition scenario, while acknowledging that a continuous transition remains possible elsewhere on the phase boundary. More recently, DMRG studies of models of hardcore bosons support a continuous transition (98, 99), though they do not estimate critical exponents or check for signatures of conformal invariance.

In ref. 95, the bosonic trivial-to-Laughlin transition theory is expressed both in a form equivalent to Eq. **9** and as its fermionic dual, a ${\text{QED}}_{3}$-Chern–Simons theory (94). Due to a conjectured “level-rank” duality (100), the theory is believed to possess an emergent SO(3) symmetry (95, 101), rotating between the boson density, creation, and annihilation operators, which go gapless at criticality. Should this critical point be described by a conformal field theory, the putative SO(3) symmetry imposes an important constraint: Since the conserved density has protected scaling dimension 2, the Cooper pair insertion operator should have the same scaling dimension (95). Remarkably, our lattice model provides a microscopic realization of this transition in which the pseudospin ${\text{SU(2)}}_{c}$ symmetry introduced in Section 1 explicitly implements this conjectured SO(3) symmetry. In fact, we exploit the pseudospin symmetry within the nonabelian slave-rotor formalism (70) to derive a bosonic SU${\left(2\right)}_{1}$ Chern–Simons theory dual to Eq. **9** (see *SI Appendix*, section 6 for details). The conserved current of this theory manifestly transforms as an SO(3) vector under pseudospin rotations, in striking agreement with the predictions presented above (95).

### 2.2. Nonzero Doping.

In this section, we turn to nonzero doping and discuss the different regions of the phase diagram of Fig. 1*A*. In particular, we describe how both insulating phases considered above naturally give rise to superconductivity upon doping, with proximity to topological criticality playing the crucial role of relegating electron excitations to high energies.^‡^ On the IQH side, we argue this occurs via the condensation of low-lying charge-2e modes, while on the CSL side we show how superconductivity arises within a parton description of the low-lying fractionalized charge carriers.

Starting from the IQH (or Phase I), the persistence of the spin gap and reduction of the charge-2e gap by topological criticality implies that doped charges enter as spin-singlet Cooper pair excitations, i.e., bound states of electrons. This can be seen from the effective theory (Eq. **9**) in the phase where *ψ* is condensed. Shifting variables a→a+A yields the coupling $2\frac{A_{0}}{2\pi }\nabla \times a$, where $\nabla \times a=\partial_{x}a_{y}-\partial_{y}a_{x}$, which implies that charges enter as vortices of the *ψ* condensate. In particular, by flux quantization ∫∇×a∈2πZ, they are forced to enter as charge-2e objects, or Cooper pairs.

Per unit cell of the triangular lattice, these pairs experience 2π external flux, twice that of their constituent electrons. Thus, magnetic translations do not enforce degenerate minima in the energy landscape of Cooper pair excitations, unlike at generic flux fractions where the Cooper pair effective mass would also be suppressed by a narrower bosonic Hofstadter bandwidth.^§^ In fact, in Section 3, we provide numerical evidence that there is a dispersive, nondegenerate 2e bound state. At U/t=6 on the 4×4 torus, in Subsection 3.1, we estimate its effective mass to be $m_{\text{Cooper}}\approx 1.3\;\hbar^{2}/ta^{2}$. While this provides an order-of-magnitude estimate of the Cooper pair effective mass deep in the IQH phase, the parton theory dictates that the effective mass vanishes on approaching the critical point, with the dispersion approaching ω∝|k|. Moreover, we expect the low-lying Cooper pairs to have a characteristic size $\ell_{c}$ controlled by the spin gap.

We now consider doping the IQH insulator. At sufficiently low dopant density compared to $\ell_{c}^{-2}$ and $\xi^{-2}$, where *ξ* is the charge-2e correlation length (*SI Appendix*, section 4), we have a dilute gas of Cooper pairs with short-range repulsive interactions and small effective mass. These are expected to enter a superfluid phase, where the superfluid density increases continuously from zero as *μ* is tuned across the IQH-superconductor transition, as in the Bose-Hubbard model away from integer filling (82, 107). At higher densities, it would be valuable to explore in detail whether longer-range bosonic interactions mediated by critical fluctuations can lead to crystallization (108) or phase separation (109).

The charge and spin response of the spin-singlet IQH insulator are $\sigma_{\mathrm{xy}}=2\cdot e^{2}/h$ and $\sigma_{\mathrm{xy}}^{s}=2\cdot \hbar /8\pi$, respectively, with edge chiral central charge $c_{-}=2$. On condensing spin-singlet Cooper pairs, the spin quantum Hall response is unaffected since the spin sector remains gapped. However, including their superfluid response removes the quantization of charge Hall conductance, while the chiral central charge is unchanged (110). This predicts edge states usually associated with the weak-pairing “d+id” superconductors (85, 111). However, as later shown in Section 3, we are far from the weak-pairing limit, so that the symmetry of the Cooper pair wavefunction is unrelated to the topological properties and edge states of the superconductor (112). Moreover, as we later discuss in Section 3.3, the terminology regarding pairing symmetry must be adapted to the present scenario of magnetic space group symmetries (60, 113).

Starting from the CSL (or Phase II), we now have fractionalized elementary excitations and must appeal to the parton description. Our analysis elucidates a remarkable connection to the mechanism of anyon superconductivity, and also the possible microscopic realization of higher-charge superconductivity in related models. We consider doping a finite charge density of 0<y≪1 holes per site, which requires that the rotor density be $\langle L_{i}\rangle =-y$. The constraint Eq. **5** correspondingly demands a depletion of spinons relative to integer filling: $\langle n_{i↑}^{f}+n_{i↓}^{f}\rangle =1-y$, which seemingly poses a problem since we expect the spin gap, and hence the spinon gap, to remain open upon light doping. Fortunately, since the spinons occupy bands with Chern number $C_{↑}=C_{↓}=1$, their density can be tuned by introducing additional gauge flux $n_{\phi }\equiv \left(\nabla \times a\right)/2\pi =-y/2$.

To evaluate the resulting physical behavior and elucidate the possibility of superconductivity, let us concentrate on hole doping and replace our rotor with a hardcore bosonic “holon” *b* on each site, with $n_{b}=b^{†}b\in \left\{0,1\right\}$, and write $c_{\sigma }^{†}=bf_{\sigma }^{†}$. The Hilbert space on each site is restricted to empty and single-electron states, which both satisfy the slave-boson constraint $n_{b}=1-n_{f}$ (76). Following our previous analysis but with the canonical boson *b* rather than the rotor, integrating out *f* leaves:

[10] $$\begin{array}{c} \begin{array}{cc} L_{\text{doped}} & =b^{*}\left(i\partial_{t}+a_{0}-A_{0}\right)b-{\left|{\vec{D}}_{a-A}b\right|}^{2}-V\left(b\right)\\ & +\frac{2}{4\pi }a\wedge da+\frac{2}{4\pi }A_{s}\wedge dA_{s}. \end{array} \end{array}$$

This theory incorporates the feature that the density of holons is constrained to be $n_{b}=-2n_{\phi }$, seen from the equation of motion for $a_{0}$. While there are a variety of possible phases of bosons at the filling $\nu_{\text{holon}}=-2$, a very natural one is the bosonic integer quantum Hall (bIQH) state with even integer Hall conductivity (114, 115), a symmetry protected topological (SPT) phase (116) (where the protecting U(1) symmetry is taken to be the gauge “symmetry”). The bIQH phase hosts symmetry-protected counterpropagating edge states and has no intrinsic topological order. Thus it is entirely captured by its effective response (114), probed here by the gauge field combination a−A, namely $\Delta L_{\text{holon}}=-\frac{2}{4\pi }\left(a-A\right)\wedge d\left(a-A\right)$. Adding this to the effective theory for Phase II we obtain the finite-doping theory in which, importantly, the dynamical Chern–Simons term cancels out:

[11] $$\begin{array}{c} L_{\text{doped}}=\frac{2}{2\pi }A\wedge da-\frac{2}{4\pi }A\wedge dA+\frac{2}{4\pi }A_{s}\wedge dA_{s}. \end{array}$$

This phase therefore lacks intrinsic topological order. It has a single bulk gapless mode due to the Maxwell dynamics of *a*, representing the Goldstone mode of the spontaneously broken ${\text{U(1)}}_{c}$ symmetry, absent *A*. From the Ioffe-Larkin rule for adding spinon and holon resistivity tensors, $\rho =\rho_{\text{spinon}}+\rho_{\text{holon}}$ (117–120), we also conclude that the fluid has vanishing resistivity (76). This is because the partons have opposite Hall responses with respect to *a*:

[12] $$\begin{array}{c} \rho_{\text{spinon}}=-\rho_{\text{holon}}=\frac{1}{2}\left(\begin{array}{cc} 0 & -1\\ 1 & 0 \end{array}\right). \end{array}$$

Furthermore, we see from the first term in Eq. **11** that a fundamental flux ∫∇×a=2π couples minimally to *A* and binds 2e charge. This is therefore a superconductor of Cooper pairs, and the monopole operator that inserts unit flux of *a* is their creation operator. In fact, Eq. **11** (with Maxwell terms for *a* and *A*) can be mapped to a Ginzburg–Landau Lagrangian for a 2D superconductor (28, 121).

The Chern–Simons term for $A_{s}$ in Eq. **11** indicates a spin quantum Hall response of $\sigma_{\mathrm{xy}}^{s}=2\cdot \hbar /8\pi$. Moreover, the edge chiral central charge is $c_{-}=2$ (*SI Appendix*, section 3). We conclude that this superconductor has precisely the same topological and response properties as the one originating from the IQH. This motivates the minimal phase diagram shown in Fig. 1*A*, where a single superconducting phase emanates from the IQH and CSL phases upon doping.

#### 2.2.1. Relation to anyon superconductivity.

The discussion above has a close correspondence to anyon (or more precisely semion) superconductivity, proposed in refs. 19–27. These works argued that a gas of charge-*e* semions, obtained for instance by doping a CSL, can realize a superconducting state. This problem can be mapped to that of bosons $b^{\prime }$ attached to *π* statistical flux, which transmutes their statistics to those of the original semions (122). In this description, the boson and flux densities are therefore related by $n_{b^{\prime }}=-2n_{\phi }$, exactly the relation satisfied by the slave bosons in Eq. **10**. The analysis following that equation provides a direct link between our long-wavelength theory of the doped CSL, derived for the Hubbard–Hofstadter model, and the effective theory of semion superconductivity, most transparently its bosonic formulation developed in ref. 122. Our analysis provides an alternative characterization of the semion superconductor, namely as a bIQH phase of the $b^{\prime }$ bosons (76).

We emphasize that a key advantage of our setup is that proximity to topological criticality in the CSL phase renders doped semions (carrying charge *e* and no spin) energetically favorable compared to electrons. It also provides renewed motivation for studying the CSL-superconductor transition, which, to our knowledge, has not been characterized in detail in the anyon superconductivity literature. We hope that the bosonic SPT perspective presented above will offer tools and insights for advancing its understanding (123, 124).

The bIQH characterization presented above also offers insight into the possible microscopic realization of charge-*Ne* superconductivity with N∈2Z. Consider electrons in the fundamental representation of a flavor SU(N) symmetry and apply a background flux of 2π/N per unit cell. At a density of one electron per site, the noninteracting phase is an IQH insulator with $\sigma_{\mathrm{xy}}=Ne^{2}/h$, where the lowest Hofstadter band is filled by all *N* flavors. On increasing *U*, we expect to realize a CSL with $\text{SU}{\left(N\right)}_{1}$ (or equivalently $\text{U}{\left(1\right)}_{N}$) topological order and chiral central charge $c_{-}=N-1$ (74). A possible critical theory is then the SU(N) analogue of Eq. **9** above, represented as a complex scalar field *χ* coupled to a U(1) gauge field with a level-*N* Chern–Simons term:

[13] $$\begin{array}{c} L=|D_{A-a}{\chi |}^{2}-V\left(\chi \right)+\frac{N}{4\pi }a\wedge d\;a. \end{array}$$

The IQH and CSL again correspond to the condensed and gapped phases for *χ*, respectively. Upon doping, superconductivity can arise if the excess bosons enter a bIQH phase, yielding a Hall contribution $\frac{M}{4\pi }a\wedge da$, where *M* is necessarily even (114). Therefore, the Chern–Simons term in Eq. **13** may be canceled out, and a conventional superconductor can arise (28), for even integer *N*. Furthermore, these correspond to a condensate of charge-*Ne* electron composites which are singlets under the SU(*N*) and clearly can only condense for even *N*. Note that the charge-*e* anyons in this scenario have statistical angle θ=π/N, distinct from π(1−1/N) in the classic anyon superconductivity scenario (22). They only agree for the special case of N=2, i.e., semion superconductivity, considered in this work.

At N=2, it is tempting to view anyon superconductivity as arising from the binding of pairs of charge-*e* semions into bosons, that then condense. We remark however that this route would leave the remaining semionic excitations deconfined, resulting in a very different fractionalized superconductor “SC^∗^” (125) that retains semionic excitations. Due to the absence of a Chern–Simons term for *a* in Eq. **11**, the anyon superconductor mechanism evidently yields a superconductor of the more conventional variety (22, 122).

## 3. Numerical Evidence for Cooper Pairing

In the preceding section, we presented field-theoretic arguments for the nature of the putative critical theory and its excitations, as well as the intriguing possibility of topological superconductivity upon doping near this critical point. Here, we provide explicit numerical evidence for an important precursor to superconductivity, electron pairing above the half-filled ground states.

### 3.1. Exact Diagonalization.

We first investigate the system using ED. We focus on 6×2 and 4×4 site systems, the latter being the largest we can access. We diagonalize the Hamiltonian at the particle–hole symmetric point μ=0, and organize the spectrum by the charge *Q* and spin $S_{z}$ quantum numbers, taking the convention where the half-filled ground state has $Q=S_{z}=0$ and zero energy. To construct the 6×2 torus, we identify points separated by the vectors $6a_{1}$ and by $2a_{2}-4a_{1}$. On the 4×4 system, we identify points related by $4a_{1}$ and $4a_{2}$ (see Fig. 1*B* for the definition of the lattice vectors $a_{1,2}$).

**Fig. 2.**
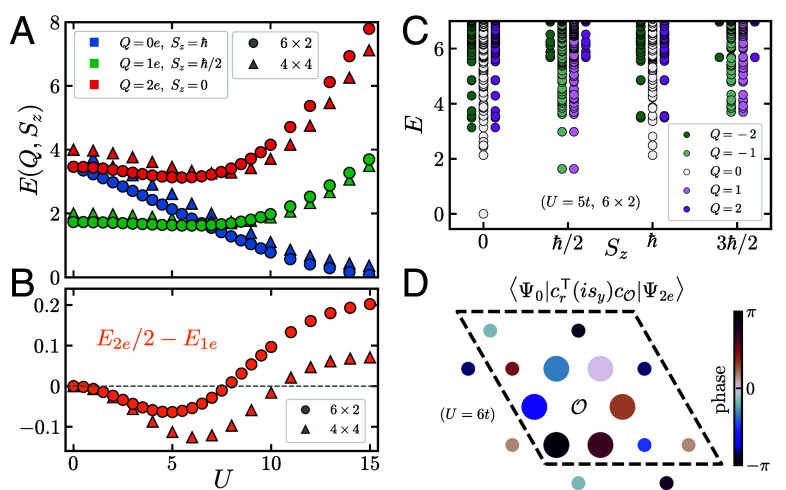
(*A*) Exact diagonalization (ED) energies of the lowest-lying states in three charge sectors (indicated in legend) for both the 4×4 site (triangular markers) and 6×2 site systems (circles). (*B*) Plot of $E_{2e}/2-E_{1e}$ vs. *U*, whose negative value indicates electron pairing. (*C*) ED energy spectrum on the 6×2 site torus at U/t=5. The energies are arranged by total spin $S_{z}$ and electric charge *Q* (indicated in legend); they organize into multiplets of the spin and pseudospin SU(2) symmetries. (*D*) Overlap of the spin-singlet pairing operator $c_{r}^{T}\left(is_{y}\right)c_{O}$ between the half-filled ground state and two-electron excitation for all sites r on a 4×4 site torus at U/t=6, with origin indicated by O. Its magnitude and phase are specified by marker area and color, respectively.

In Fig. 2*A*, we plot the energies of the lowest-lying excited states in the charge sectors $\left(Q,S_{z}\right)$ specified by {(0e,ħ),(1e,ħ/2),(2e,0)}, for both the 6×2 and 4×4 systems. While we focus on the electron-doped side, adding holes is energetically equivalent under particle–hole symmetry. The lowest 0e excitation energy decreases monotonically as a function of *U*. On the other hand, both 1e and 2e exhibit minima at intermediate coupling: For the 6×2 system, these occur at $U_{1e}^{\text{min}}/t≃6.0$ and $U_{2e}^{\text{min}}/t≃5.5$, whereas on the 4×4 torus these values increase to $U_{1e}^{\text{min}}/t≃8$ and $U_{2e}^{\text{min}}/t≃7$.

The ED calculation convincingly demonstrates that the lowest-lying charge excitations are paired over a remarkably broad range of interaction strengths. Specifically, the energy $E_{2e}$ of the lowest-lying charge-2e state is less than that of two individual electronic excitations, i.e., $E_{2e}-2E_{1e}<0$. We plot this quantity in Fig. 2*B*, which we find to be negative in the range 0<U/t≤7.5 for the 6×2 system and over an even broader range 0<U/t≤10 for the larger 4×4 system. The maximum magnitude of the pairing energy increases from 0.06t to 0.13t between the two systems, constituting 4% and 7% of the corresponding 1e energies, respectively. The location of this maximum is $U_{\text{max}}/t≃5$ for the 6×2 system, increasing to $U_{\text{max}}/t≃6$ on the larger 4×4 torus. Though these features occur at smaller interaction strength than the expected IQH-CSL transition point U/t≈12 reported by previous cylinder iDMRG studies (49, 50), we anticipate that $U_{\text{max}}$ will continue to increase with increasing system size.

The numerical observation of pairing is important, confirming evidence for topological criticality and the associated softening of charge-2e modes. Moreover, as argued in Section 2.2, the existence of electron pairing in the IQH phase is a direct precursor to superconductivity and is likewise expected in the CSL near criticality. To estimate the Cooper pair effective mass, crucial to phase stiffness, we thread small flux *φ* through the torus and compute the change in the charge-2e energy $E_{2e}$. For concreteness, we fix U/t=6 on the 4×4 torus, where pairing is maximal, though on larger systems we expect this point to reside deep in the IQH phase (see refs. 49 and 50). At small *φ*, we obtain a quadratic fit $E_{2e}\left(\varphi \right)/t\approx 5.01{\left(\varphi /2\pi \right)}^{2}$. Threading 2π flux shifts the total two-electron momentum q by 2/L times a primitive reciprocal vector of magnitude $4\pi /a\sqrt{3}$, where L=4 is the length of the torus. Matching the dispersion to $\hbar^{2}q^{2}/2m_{\text{Cooper}}$ with $\varphi /2\pi =qa\sqrt{3}/2\pi$, we estimate $m_{\text{Cooper}}\approx 1.3\;\hbar^{2}/ta^{2}$ at the chosen system size and interaction strength, providing an order-of-magnitude estimate for the pair effective mass in the IQH phase.

We note that the pairing energy $E_{2e}/2-E_{1e}$ cannot be positive in the thermodynamic limit because it is always possible to create a well-separated pair of 1e excitations with energy equal to that of independent electrons. Its taking positive values for U/t>7.5 and U/t>10 on the 6×2 and 4×4 systems, respectively, is therefore a finite size effect. Given that the interactions are repulsive, the observed (negative) electron pairing energy at smaller *U* has no similarly compelling finite-size explanation, thus pointing to topological criticality as its origin. Furthermore, the fact that pairing extends down to seemingly arbitrarily small values (probed down to $U/t=10^{-3}$) suggests a weak-coupling origin complementary to pairing induced strictly by proximity to criticality, which we further elucidate in Section 3.4 by a small-*U* perturbative calculation on much larger systems.

Each of the systems under consideration possesses the SO(4) symmetry explicated in Section 1. Accordingly, we observe that the ED spectrum organizes into multiplets of the spin and pseudospin SU(2) symmetries. This is displayed in Fig. 2*C* for the 6×2 torus at U/t=5, in which eigenstates with fixed spin $S_{z}$ but different total charge *Q* populate multiplets of the pseudospin ${\text{SU(2)}}_{c}$. For both system sizes, the $\left(Q,S_{z}\right)=\left(0e,\hbar \right)$ state in Fig. 2*A* belongs to a pseudospin-singlet spin-triplet irreducible representation, while the $\left(Q,S_{z}\right)=\left(2e,0\right)$ branch is a pseudospin-triplet spin-singlet.

We remark that pairing was not observed on the smaller 4×2 torus, nor on the 6×2 system with the identification $r∼r+2a_{2}∼r+6a_{1}$, though we recall that pairing was observed at 6×2 under the boundary identification specified earlier. Nonetheless, it is suggestive that pairing is present on the largest accessible system, the 4×4 torus studied above. Verifying pairing at larger system sizes, employing approximate methods beyond ED, is an important task for future work. In the next section, we take one step forward by establishing electron pairing in a cylindrical geometry of finite width but infinite length using tensor network methods.

### 3.2. Segment DMRG.

Here, we employ the “infinite boundary condition” technique (126–129), referred to henceforth as the “segment” DMRG method (127, 130, 131, 169), to obtain excited states and their energies. Starting from an infinite MPS approximation to the ground state at half-filling, we allow the tensors of the excited state MPS to differ on a segment spanning $N_{r}$ cylinder rings. DMRG is then used within this variational class to minimize the excitation energy for fixed quantum numbers $Q,S_{z}$, and circumferential momentum $k_{y}$ relative to the ground state. The segment DMRG method lets us probe finite-charge excitations above the half-filled ground state without introducing a physical edge.

**Fig. 3.**
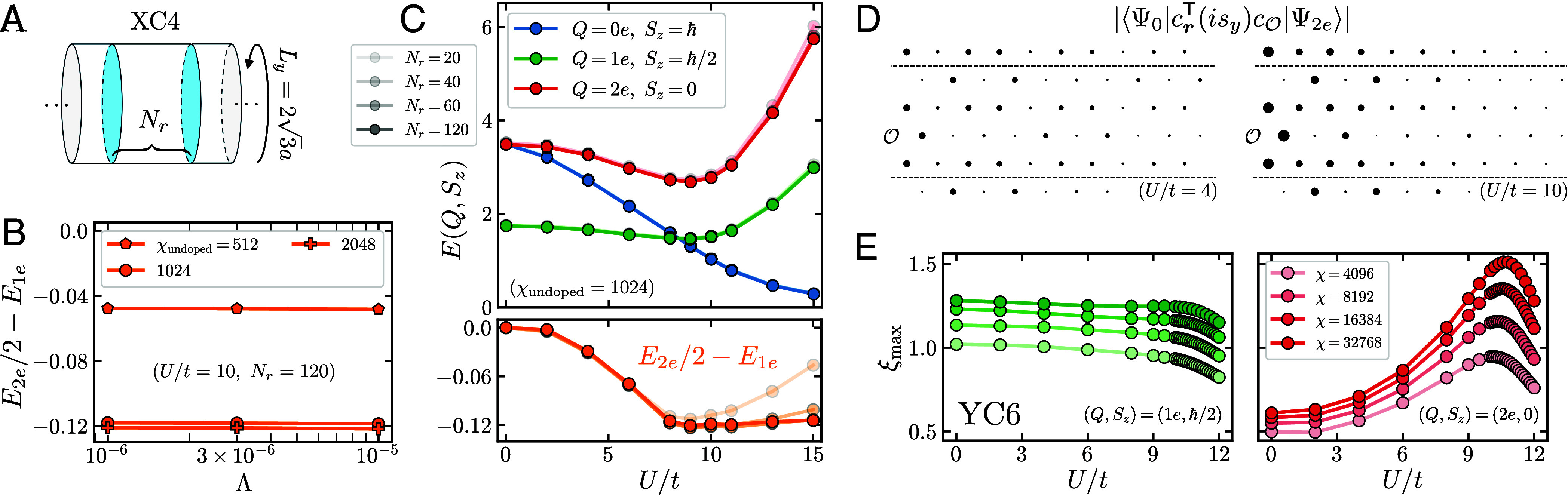
(*A*) Illustration of the infinite-length XC4 cylinder with circumference $L_{y}=2\sqrt{3}a$, where *a* is the lattice spacing. The “segment” DMRG method consists of optimizing a finite number of tensors (describing $N_{r}$ cylinder rings) sandwiched between two semi-infinite MPS environments derived from a reference (half-filled) ground state with bond dimension $\chi_{\text{undoped}}$. (*B*) Electron pairing energy $E_{2e}/2-E_{1e}$ vs. maximum magnitude Λ of discarded singular values in the finite segment, with legend indicating $\chi_{\text{undoped}}$. (*C*) *Upper* panel: energies of excitations with charges $\left(Q,S_{z}\right)$ (indicated in legend) vs. Hubbard *U*, with fixed $\chi_{\text{undoped}}=1,024$ and $\Lambda =10^{-5}$. The legend indicates $N_{r}$ (increasing light to dark). *Lower* panel: electron pairing energy vs. *U*. (*D*) Magnitude of the pair wavefunction at both U/t=4 and U/t=10, computed from respective iDMRG ground states with $\chi_{\text{undoped}}=1,024$ and segment excitations with $N_{r}=120$. The two dotted lines are related under the cylinder identification; outside points are periodic images. (*E*) Correlation lengths of the half-filled YC6 ground state in the 1e (*Left* panel) and 2e (*Right*) sectors, in units of cylinder rings, as a function of bond dimension (increasing light to dark) and *U*.

For the XC4 cylinder considered here and shown schematically in Fig. 3*A*, we identify points separated by $4a_{2}-2a_{1}\propto \hat{y}$ around the circumferential direction (with circumference $2\sqrt{3}a$), but take the cylinder to have infinite length, with $a_{1}$ parallel to the cylinder axis (30). Each cylinder ring consists of two spinful lattice sites. We employ a gauge in which the magnetic unit cell consists of two sites, with magnetic Bravais vectors given by $a_{1}$ and $2a_{2}-a_{1}$ (*SI Appendix*, section 1C). This results in two distinct momenta $k_{y}$, which label the eigenvalues of the circumferential translation ${\left(T_{2}\right)}^{2}T_{1}^{†}$. To obtain the parent half-filled states, we use the iDMRG algorithm (132–134), and conserve the charges $\left(Q,S_{z},k_{y}\right)$ for both the ground state and the segment excitation calculations (131, 169).

Accurate results require converging in three separate parameters: the bond dimension $\chi_{\text{undoped}}$ of the half-filled ground state, the bond dimension of tensors within the variational segment—which we parameterize by the maximum magnitude Λ of the discarded singular values—and the number of cylinder rings $N_{r}$ in the segment. In Fig. 3*B*, we plot the pairing energy (for U/t=10 and $N_{r}=120$ rings) as a function of Λ and parent state bond dimension. The energy is found to depend more strongly on the latter but shows little quantitative difference between $\chi_{\text{undoped}}=1,024$ and 2,048, permitting us to choose the less expensive option at other interactions *U*. We focus on the XC4 cylinder because it is the largest system on which we can obtain ≲0.01t error in the energy using available resources, and because the smaller YC3 cylinder does not exhibit pairing, presumably due to insufficient cylinder width.

In Fig. 3*C*, we plot the excitation energies in the charge sectors $\left(Q,S_{z}\right)$ given by {(0e,ħ),(1e,ħ/2),(2e,0)} as a function of Hubbard interaction *U* and the segment length $N_{r}$ (increasing with shading, light to dark), fixing $\chi_{\text{undoped}}=1,024$. For each $\left(Q,S_{z}\right)$, we initialize in each momentum sector $k_{y}$ and take the minimum energy. We remark that the energy curves bear strong resemblance to those obtained from ED in Fig. 2*A*. For the DMRG, we observe that the smaller-*U* simulations exhibit more rapid energetic convergence in $N_{r}$. Both the 1e and 2e energies monotonically decrease from U/t=0 to a minimum at U/t≈9 and increase rapidly thereafter. Consistent with ED, their energies indicate electron pairing $E_{2e}/2-E_{1e}<0$ over a broad range of interaction strengths, from U=0 to at least U/t=15. The maximum pairing strength of 0.12t occurs at U/t=9, near the putative critical point discussed in Section 2.1, constituting 8.3% of the corresponding 1e energy, though the data in fact exhibit a plateau in the pairing energy beginning at this *U*.

The behavior of the charge-neutral $S_{z}=\hbar$ excitation branch (Fig. 3*C*) strongly resembles the corresponding energy curve in ED, where we identified it as belonging to a spin-triplet, pseudospin-singlet representation. In the segment DMRG data, this excitation energy decreases gradually from its expected value of $2\sqrt{3}t$ at U=0 to zero at U/t>15. This agrees with the results from ED for both the 6×2 and 4×4-site systems, shown in Fig. 2*A*, where this gap similarly closes at U/t>15. Agreement between these three geometries suggests this feature persists in the thermodynamic limit, where it may be associated with a transition to the 120^°^ antiferromagnetic phase, setting an upper-bound on the extent of the putative CSL phase.

Though our explicit DMRG pairing calculations are limited to the XC4 cylinder, indirect evidence for the persistence and possible enhancement of pairing on wider cylinders is provided by the behavior of the transfer matrix spectrum associated with the translation-invariant half-filled parent state (49, 50). The dominant transfer matrix eigenvalues, resolved by charge sector $\left(Q,S_{z},k_{y}\right)$, relate to ground state connected correlation lengths of operators carrying those charges (135, 136). In the $\text{YC-}L_{y}$ cylinder sequence (30), the correlation length associated with spin-singlet 2e excitations becomes more pronounced as $L_{y}$ increases from $L_{y}=3$ to 6 (*SI Appendix*, section 4). In Fig. 3*E*, we showcase the 2e and 1e correlation lengths on the YC6 cylinder, where the pronounced peak of $\xi_{2e}$ and its prominence over $\xi_{1e}$ suggests the persistence of electron pairing on this wider cylinder. The same pattern holds when comparing the XC4 and XC6 cylinders. In all cases, by threading flux if necessary, we ensure that the fluxes penetrating the cylinder rings are consistent with the particle–hole SO(4) symmetry of Section 1.

### 3.3. Pairing Symmetry.

In this section, we discuss the symmetry of the charge-2e paired states obtained in ED and DMRG. We argue that these low-energy excitations are spin-singlet and energetically nondegenerate, carrying odd angular momentum under site-centered rotations and even angular momentum under bond-centered rotations.

On the 4×4 system studied in ED, the uniqueness of the lowest-energy charge-2e excited state for all *U* implies that it is spin-singlet and transforms in a one-dimensional irreducible representation of the magnetic space group generated by $T_{1},C_{6}$. We find the pair carries momentum $T_{j}|\Psi_{2e}\rangle =-|\Psi_{2e}\rangle$ for all nearest-neighbor translations $T_{j}$. Counterintuitively, this is in fact the unique momentum which is rotation-symmetric: Invariance under $C_{6}$ requires each $T_{j}$ to have the same eigenvalue, while Eq. **4** requires $T_{5}T_{3}T_{1}=-1$ when acting on a pair, which together imply $T_{j}=-1$. Moreover, we find that $C_{2}|\Psi_{2e}\rangle =-|\Psi_{2e}\rangle$, so that the state has odd angular momentum with respect to site-centered rotations. However, for each bond-centered rotation $C_{2}^{\text{bond}}=T_{j}C_{2}$, we therefore have $C_{2}^{\text{bond}}|\Psi_{2e}\rangle =+|\Psi_{2e}\rangle$, which has invariant meaning since ${\left(C_{2}^{\text{bond}}\right)}^{2}=1$ in every fermion number sector. Thus, the pair has even angular momentum with respect to all bond-centered rotations.^¶^

By antisymmetry of the wavefunction, spin-singlet pairing should therefore be allowed between sites related by $C_{2}^{\text{bond}}$ but disallowed when they are related by a site-centered rotation $C_{2}^{\text{site}}$. This is exactly borne out in the 4×4 ED numerical data. In Fig. 2*D*, we plot the spin-singlet “pair wavefunction” $\left\langle \Psi_{0}|c_{r}^{T}\left(is_{y}\right)c_{O}|\Psi_{2e}\right\rangle$ at U/t=6, where $\Psi_{0}$ is the ground state, O is the origin, and $c^{T}$ denotes the transpose of the column vector of electron annihilation operators. We observe that the wavefunction vanishes when r is related to O by some $C_{2}^{\text{site}}$, in agreement with the above arguments. Moreover, the pair is well localized, with nearest-neighbor pairing having much larger magnitude than next-nearest-neighbor pairing. Though the phase winding in Fig. 2*D* suggests the pairing symmetry is “p+ip,” we caution that the $C_{3}$ eigenvalue of the charge-2e excited state does not have obvious invariant meaning as it can be modified by the redefinition $C_{3}\to {\left(e^{2\pi i/3}\right)}^{N_{F}}C_{3}$. Nonetheless, on the 4×4 torus, we have unambiguously confirmed spin-singlet pairing, carrying odd (even) angular momentum under site-centered (bond-centered) rotations.

On the XC4 cylinder, three-fold rotation symmetry is explicitly broken. Nonetheless, the segment DMRG pair excitations exhibit the same pairing symmetry as above. We verify this explicitly by computing the pair wavefunction, choosing O at the center of the segment.^#^ We find it is odd under $r\to C_{2}r$ and that the spin-triplet overlap is several orders of magnitude smaller than spin-singlet. Moreover, as shown in Fig. 3*D* for U/t=4 and 10, we find that the pair wavefunction vanishes approximately (error is induced by finite $N_{r}$) when r and O are related by some $C_{2}^{\text{site}}$, which indicates $C_{2}^{\text{site}}|\Psi_{2e}\rangle =-|\Psi_{2e}\rangle$. Similarly, we conclude that $C_{2}^{\text{bond}}|\Psi_{2e}\rangle =+|\Psi_{2e}\rangle$. These plots also show that the size of the excitation decreases with *U*. Finally, since we conserve momentum around the cylinder, our excited states are labeled by their eigenvalue under the circumferential translation $T={\left(T_{2}\right)}^{2}T_{1}^{†}$. For all *U*, the paired eigenstate satisfies $T|\Psi_{2e}\rangle =-|\Psi_{2e}\rangle$, consistent with our expectation $T_{1}=T_{2}=-1$.

We anticipate that our pairing symmetry results will help guide future numerical investigations of pairing and superconductivity in this model, especially those employing techniques that operate within a fixed charge sector, such as the ED and DMRG performed in this work.

### 3.4. Pairing at the Conduction Band Edge.

Both our ED (Fig. 2) and DMRG (Fig. 3) numerics indicate that pairing extends down to small values of *U*. Although our analytical discussion has focused largely on the intermediate-coupling regime near topological criticality, the regime of perturbatively weak interactions allows for an independent check of the existence and symmetry of bound pairs in our model, which we fix here to be spin-singlet. To second order in many-body perturbation theory, renormalization of the two-body scattering vertex is described by the following five diagrams (137–139):[14]
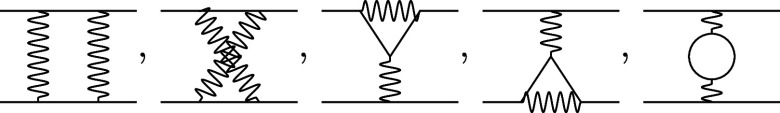


where curvy and straight lines denote interaction events and single-particle propagators, respectively. As shown in ref. 140 in the context of superconductivity mediated by local repulsion (141, 142), some of these diagrams vanish given a specific bare interaction kernel. For our model at one electron per site and zero temperature, we show in *SI Appendix*, section 7 that only the “cross” diagram contributes to the Cooper vertex in the spin-singlet channel. Restricting our attention to the four degenerate single-particle states at the conduction band edge, labeled by their momenta $\kappa ,\kappa^{\prime }$ and degenerate band index n∈{1,2} (Fig. 1*C*), we find that this diagram only mediates pairing in the channel

[15] $$\begin{array}{c} \begin{array}{cc} \hat{\Delta } & =c_{\kappa^{\prime },↑,2}c_{\kappa ,↓,1}-c_{\kappa^{\prime },↓,2}c_{\kappa ,↑,1}\\ & -c_{\kappa ,↑,2}c_{\kappa^{\prime },↓,1}+c_{\kappa ,↓,2}c_{\kappa^{\prime },↑,1}. \end{array} \end{array}$$

Consistent with ED and DMRG, this spin-singlet pair has odd angular momentum with respect to site-centered rotations.

## 4. Discussion

A central message of this work is that topological superconductivity can emerge in a regime with both strong repulsive interactions and broken time reversal symmetry. Electron pairing was investigated and was clearly observed in our numerical calculations in the present Hofstadter–Hubbard model. It is strongest in the regime of intermediate interaction strength close to the putative IQH-CSL critical point (49, 50) and remarkably remains nonzero down to perturbatively weak interactions. The pairs are spin-singlet, with odd (even) angular momentum under site-centered (bond-centered) rotations.

These numerical observations support the principal idea that proximity to topological quantum criticality can offer a robust route to superconductivity, even in remarkably simple settings. The crucial ingredient here is that the topological transition is associated with the closing of the charge gap, while the spin gap remains open. From the IQH-CSL critical theory formulated for the present Hofstadter–Hubbard model, we argued that doping naturally leads to a topological superconductor. On the CSL side of the phase diagram, this relates to the storied mechanism of semion superconductivity. However, given the broad extent around criticality where superconductivity is anticipated, it is not strictly necessary for this mechanism that the topological critical point or the CSL be accessed in a given model. For instance, superconductivity could emerge upon doping the IQH well away from criticality, even in a scenario where the CSL is entirely subsumed by a conventional magnetically ordered phase, or if the transition exists but is weakly first-order. Likewise, we do not expect $C_{2}$ breaking or deviation from particle–hole symmetry to play a significant role away from the weak-coupling regime.

We now list key areas for future exploration. A more complete characterization of the quantum critical point and its associated conformal field theory would further elucidate the likely universal nature of superconducting onset upon doping the IQH, CSL, and the critical point itself. It may also shed light on potential competition with conventional (108) and fractionalized (143, 144) crystalline phases at nonzero doping. Another immediate next step is numerically verifying the existence of the superconducting ground state in this model, for instance with cylinder DMRG (145–148) or other suitable methods. Furthermore, to distinguish features unique to our model from those of the general theoretical scenario near criticality, it will be necessary to compare with a broader class of models that relax various symmetries. Such results should also be compared to existing numerical observations of superconductivity in related models on the triangular lattice, namely chiral *t*-*J* models with real hoppings (149–152), where the charge fluctuations integral to topological criticality are absent at half filling.

Next, we highlight promising experimental realizations in moiré materials. Triangular lattices formed by TMD sheets with a moiré potential offer both valley and layer “pseudospin” degrees of freedom (153–158). As proposed in refs. 61 and 49, the Hofstadter–Hubbard model considered here could be realized by applying a perpendicular magnetic field to such a system with a tens-of-nanometer moiré lattice constant. The field would conveniently polarize the valley (i.e., true spin) degree of freedom due to the Zeeman effect, yielding the effective model of Section 1, with layer pseudospin playing the role of spin. Further numerical studies of this model and its deformations would help guide the experimental exploration of criticality and superconductivity in these moiré platforms.

Finally, the recent observation of the fractional quantum anomalous Hall effect in twisted bilayer ${\text{MoTe}}_{2}$ (159–162) and rhombohedral multilayer graphene (163), as well as fractional Chern insulators in both magic-angle graphene at weak magnetic field (164) and in the field-induced Chern bands of Bernal bilayer graphene aligned with hexagonal boron nitride (165), motivate exploring the behavior of charged anyons subject to substantial lattice effects, as we have in this work. Further, a recent experiment on quadrilayer rhombohedral graphene demonstrates that chiral superconductivity is possible even at strong magnetic fields (166), a hopeful sign for the class of mechanisms proposed here, motivating the study of routes to pairing and superconductivity in the strong time-reversal-breaking regime.

### 4.1. Note Added.

As this manuscript was being completed, refs. 167 and 168 appeared, which share some overlap with the present work.

## Supplementary Material

Appendix 01 (PDF)

## Data Availability

Tensor network calculations were performed using the TeNPy Library (131, 169). All study data are included in the article and/or *SI Appendix*.

## Footnotes

^*^This contrasts with the scenario of electrons at zero magnetic field (76, 77), where the two parton species experience opposite, nonzero net flux.

^†^While resembling the Zhang–Hansson–Kivelson composite boson description of the FQHE (83, 84), Eq. **9** differs in its Lorentz-invariance, particle–hole symmetry $\left(\psi ,a\right)\to \left(\psi^{*},-a\right)$ absent the probe gauge fields, and integer-level dynamical Chern–Simons term.

^‡^This distinguishes our mechanism from those invoking proximity to a critical point in a metal (102), where a fluctuating order parameter field is commonly proposed to induce electron pairing (103).

^§^The condensation of interacting Hofstadter bosons has been established even at smaller flux fractions (104–106).

^¶^All other bond-centered two-fold rotations are related by the square of some translation operator and thus have the same eigenvalue.

^#^The inversion center O is approximate since the segment contains a finite, even number of rings.
