# Supplementary material for: Anyon superconductivity from topological criticality in a Hofstadter–Hubbard model
Source: Proc Natl Acad Sci U S A. 2025 Aug 12;122(33):e2426680122. doi: 10.1073/pnas.2426680122 (PMC12377739; doi:10.1073/pnas.2426680122)
Supplement: Supplementary file 1 — Appendix 01 (PDF) [file pnas.2426680122.sapp.pdf]

Supporting Information for

Anyon Superconductivity from Topological Criticality in a Hofstadter-Hubbard Model

Stefan Divic, Valentin Crépel, Tomohiro Soejima (副島智大), Xue-Yang Song, Andrew Millis, Michael P. Zaletel, Ashvin Vishwanath

To whom correspondence should be addressed. E-mail: stefan_divic@berkeley.edu and avishwanath@g.harvard.edu

This PDF file includes:

Supporting text
Figs. S1 to S3
SI References

Supporting Information Text

Contents

1 Coordinates, Gauge Choice and Symmetries 2
A Triangular lattice coordinates . . . 2
B Imaginary C_6 gauge . . . 2
B.1 Closed-form composition of translation generators . . . 4
C C_2 gauge for XC geometries . . . 5

2 Details of the U(1) Slave Rotor Theory and Mean Field Estimate of the Transition Point 5

3 Chirality of Edge States from K-Matrix Formalism 6

4 Correlation Lengths at Half-Filling from Cylinder iDMRG 8

5 Spin and Pseudospin SU(2) Formalism 8
A Relation between spin and pseudospin generators . . . 8
B Fermionic matrix structure in $\mathrm{SU}(2)_s \times \mathrm{SU}(2)_c$ formalism . . . 11
C Hofstadter-Hubbard Hamiltonian . . . 12

6 SU(2) Slave-Rotor Theory of IQH-CSL transition 12
A Physical Hilbert space from parton constraint . . . 13
B Functional integral representation . . . 13
C Low-energy $\mathrm{SU}(2)_1$ Higgs-Chern-Simons theory . . . 14
D Conserved current . . . 15

7 Electron Pairing from Small-U Diagrammatic Expansion 16
A Band basis in the doubly-folded Brillouin zone . . . 16
B Perturbative demonstration of odd-angular momentum spin-singlet pairing . . . 17

1. Coordinates, Gauge Choice and Symmetries

A. Triangular lattice coordinates. Here, we specify the Bravais vectors of the underlying triangular lattice (see Fig. 1(b) of the main text):

$$\boldsymbol{a}_1 = \hat{x}, \qquad \boldsymbol{a}_2 = \frac{\hat{x}}{2} + \frac{\hat{y}\sqrt{3}}{2} \tag{1}$$

where we've set the nearest-neighbour lattice bond length to unity, $a = 1$. The lattice points are then given by

$$\boldsymbol{r} = n_1\boldsymbol{a}_1 + n_2\boldsymbol{a}_2, \qquad n_i \in \mathbb{Z}, \tag{2}$$

We take the reciprocal lattice vectors to be

$$\boldsymbol{b}_1 = 2\pi\hat{x} - \frac{2\pi}{\sqrt{3}}\hat{y}, \qquad \boldsymbol{b}_2 = \frac{4\pi}{\sqrt{3}}\hat{y}. \tag{3}$$

B. Imaginary C_6 gauge. Fig. S1(a) provides an electronic gauge in which the hoppings are purely imaginary *and* in which the C_6 symmetry acts in its bare form in real space:

$$C_6 c^\dagger_{\boldsymbol{r}} C_6^{-1} = c^\dagger_{C_6\boldsymbol{r}}, \tag{4}$$

provided that the (infinite 2D or torus) geometry is such that C_6 is well-defined. Thinking of a torus as a set of equivalence classes of points on the infinite plane, a well-defined spatial operation must map between equivalence classes. For example, by direction inspection, one can see that C_6 is ill-defined on the 4×2 torus.

By inspecting the phases of the hoppings in Fig. S1(a), we read off

$$T_1 c^\dagger_{\boldsymbol{r}} T_1^{-1} = i(-1)^{n_1+n_2} c^\dagger_{\boldsymbol{r}+\boldsymbol{a}_1}, \qquad T_2 c^\dagger_{\boldsymbol{r}} T_2^{-1} = i(-1)^{n_2} c^\dagger_{\boldsymbol{r}+\boldsymbol{a}_2}, \tag{5}$$

where $\boldsymbol{r} = n_1\boldsymbol{a}_1 + n_2\boldsymbol{a}_2$ is the lattice point before the transformation. To see the former, note that only the hoppings parallel to $\boldsymbol{a}_1 - \boldsymbol{a}_2$ are left invariant by bare T_1, *i.e.*, the hoppings in the $\boldsymbol{a}_1$ and $\boldsymbol{a}_2$ directions pick up a minus sign that needs to be corrected. For the latter, bare T_2 instead negates the hoppings in the $\boldsymbol{a}_2$ and $\boldsymbol{a}_1 - \boldsymbol{a}_2$ directions. The factors i are chosen so that two-fold translation is a pure shift:

$$(T_j)^2 c^\dagger_{\boldsymbol{r}} (T_j)^{-2} = c^\dagger_{\boldsymbol{r}+2\boldsymbol{a}_j}. \tag{6}$$

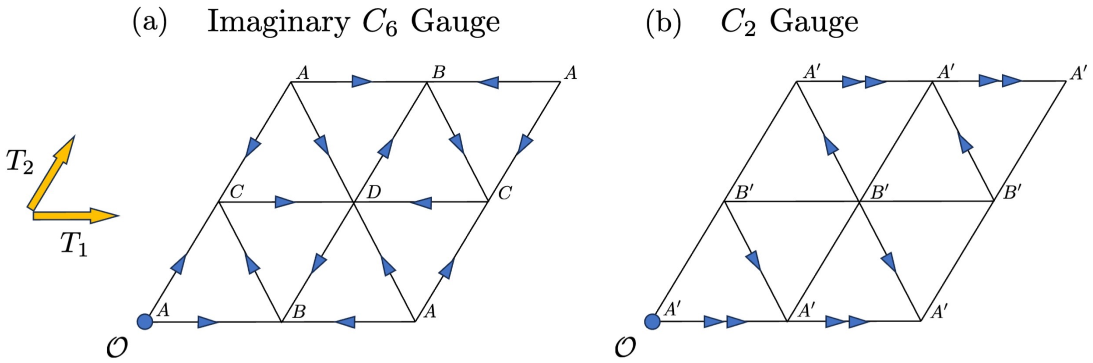

Fig. S1. Two gauge choices for the hopping Hamiltonian, in both cases giving rise to $\Phi_\triangle = \pi/2$ magnetic flux per triangular plaquette. (a) Hoppings in the "Imaginary C_6" gauge, which is manifestly C_6 symmetric about the origin [bottom left marked by $\mathcal{O}$]. The origin is chosen to coincide with the A sublattice (other sublattices B, C, D are also marked). Each arrow indicates a Peirel's phase of $+i$ in the direction of the arrow. This gauge choice is identical to that displayed in Fig. 1(b) of the main text, except there the origin is in the middle of the image. (b) Hoppings in the "C_2" gauge. All hoppings are either imaginary or real, with a single arrow again indicating a Peirel's phase of $+i$ in the direction of the arrow. There are two magnetic sublattices, A' and B'. For both (a) and (b): T_1 and T_2 (yellow arrows) denote translation symmetry in the unit directions $\boldsymbol{a}_1$ and $\boldsymbol{a}_2$, respectively. In App. 1B we provide the explicit form of T_1 and T_2 in the Imaginary C_6 gauge.

But more importantly, the definitions Eq. (4) and Eq. (5) are chosen to be consistent with all the algebraic conditions laid out in Sec. 1 of the main text.

As a non-trivial check of correctness of these translations, we can show that they correctly anti-commute:

$$(T_1 \circ T_2) \cdot c_{\boldsymbol{r}}^\dagger = -T_1 T_2 c_{\boldsymbol{r}}^\dagger T_2^{-1} T_1^{-1} \quad [7]$$
$$= -(-1)^{n_2} T_1 c_{\boldsymbol{r}+\boldsymbol{a}_2}^\dagger T_1^{-1} \quad [8]$$
$$= (-1)^{n_2} (-1)^{n_1+n_2} c_{\boldsymbol{r}+\boldsymbol{a}_2+\boldsymbol{a}_1}^\dagger \quad [9]$$

compared to

$$(T_2 \circ T_1) \cdot c_{\boldsymbol{r}}^\dagger = -T_2 T_1 c_{\boldsymbol{r}}^\dagger T_1^{-1} T_2^{-1} \quad [10]$$
$$= -(-1)^{n_1+n_2} T_2 c_{\boldsymbol{r}+\boldsymbol{a}_1}^\dagger T_2^{-1} \quad [11]$$
$$= -(-1)^{n_1+n_2} (-1)^{n_2} c_{\boldsymbol{r}+\boldsymbol{a}_1+\boldsymbol{a}_2}^\dagger, \quad [12]$$

so that indeed $T_2 T_1 = (-1)^{N_F} T_1 T_2$.

The phases can be written in a more geometric form:

$$T_1 c_{\boldsymbol{r}}^\dagger T_1^\dagger = i e^{i\boldsymbol{r}\cdot(\boldsymbol{b}_1+\boldsymbol{b}_2)/2} c_{\boldsymbol{r}+\boldsymbol{a}_1}^\dagger, \qquad T_2 c_{\boldsymbol{r}}^\dagger T_2^\dagger = i e^{i\boldsymbol{r}\cdot\boldsymbol{b}_2/2} c_{\boldsymbol{r}+\boldsymbol{a}_2}^\dagger. \quad [13]$$

From these, we can also obtain

$$(T_1^\dagger T_2) c_{\boldsymbol{r}}^\dagger (T_1^\dagger T_2)^\dagger = e^{-i\boldsymbol{r}\cdot\boldsymbol{b}_1/2} c_{\boldsymbol{r}-\boldsymbol{a}_1+\boldsymbol{a}_2}^\dagger, \quad [14]$$

and by inverting the above three,

$$T_1^\dagger c_{\boldsymbol{r}}^\dagger T_1 = i e^{-i\boldsymbol{r}\cdot(\boldsymbol{b}_1+\boldsymbol{b}_2)/2} c_{\boldsymbol{r}-\boldsymbol{a}_1}^\dagger \quad [15]$$
$$T_2^\dagger c_{\boldsymbol{r}}^\dagger T_2 = i e^{-i\boldsymbol{r}\cdot\boldsymbol{b}_2/2} c_{\boldsymbol{r}-\boldsymbol{a}_2}^\dagger \quad [16]$$
$$(T_1^\dagger T_2)^\dagger c_{\boldsymbol{r}}^\dagger (T_1^\dagger T_2) = -e^{i\boldsymbol{r}\cdot\boldsymbol{b}_1/2} c_{\boldsymbol{r}+\boldsymbol{a}_1-\boldsymbol{a}_2}^\dagger. \quad [17]$$

From this we learn that $C_6 T_1 C_6^\dagger = T_2$:

$$(C_6 T_1 C_6^\dagger) c_{\boldsymbol{r}}^\dagger (C_6 T_1 C_6^\dagger)^\dagger = (C_6 T_1) c_{C_6^{-1}\boldsymbol{r}}^\dagger (C_6 T_1)^\dagger \quad [18]$$
$$= i C_6 e^{i C_6^{-1}\boldsymbol{r}\cdot(\boldsymbol{b}_1+\boldsymbol{b}_2)/2} c_{C_6^{-1}\boldsymbol{r}+\boldsymbol{a}_1}^\dagger C_6^\dagger \quad [19]$$
$$= i e^{i\boldsymbol{r}\cdot C_6(\boldsymbol{b}_1+\boldsymbol{b}_2)/2} c_{\boldsymbol{r}+C_6\boldsymbol{a}_1}^\dagger \quad [20]$$
$$= i e^{i\boldsymbol{r}\cdot\boldsymbol{b}_2/2} c_{\boldsymbol{r}+\boldsymbol{a}_2}^\dagger \quad [21]$$
$$= T_2 c_{\boldsymbol{r}}^\dagger T_2^\dagger. \quad [22]$$

Similarly, it can be shown that $T_3 = C_6 T_2 C_6^\dagger = (-i)^{N_F} T_1^\dagger T_2$ and $C_6 T_3 C_6^\dagger = T_1^\dagger$, which is altogether consistent with $C_2 T_j C_2 = (T_j)^\dagger$ for all j.

B.1. Closed-form composition of translation generators. Here we provide the closed form expression for powers of T_1 and T_2, namely

$$T_2^M c_{\boldsymbol{r}}^\dagger T_2^{-M} = i^M c_{\boldsymbol{r}+M\boldsymbol{a}_2}^\dagger \prod_{j=0}^{M-1} e^{i(\boldsymbol{r}+j\boldsymbol{a}_2)\cdot\boldsymbol{b}_2/2} \quad [23]$$
$$= i^M c_{\boldsymbol{r}+M\boldsymbol{a}_2}^\dagger e^{i\sum_{j=0}^{M-1}\boldsymbol{r}\cdot\boldsymbol{b}_2/2} e^{i\pi\sum_{j=0}^{M-1} j} \quad [24]$$
$$= i^M c_{\boldsymbol{r}+M\boldsymbol{a}_2}^\dagger e^{iM\boldsymbol{r}\cdot\boldsymbol{b}_2/2} e^{i\pi M(M-1)/2} \quad [25]$$
$$= c_{\boldsymbol{r}+M\boldsymbol{a}_2}^\dagger e^{iM\boldsymbol{r}\cdot\boldsymbol{b}_2/2} e^{i\pi M^2/2}, \quad [26]$$

and similarly

$$T_1^M c_{\boldsymbol{r}}^\dagger T_1^{-M} = i^M c_{\boldsymbol{r}+M\boldsymbol{a}_1}^\dagger \prod_{j=0}^{M-1} e^{i(\boldsymbol{r}+j\boldsymbol{a}_2)\cdot(\boldsymbol{b}_1+\boldsymbol{b}_2)/2} \quad [27]$$
$$= i^M c_{\boldsymbol{r}+M\boldsymbol{a}_1}^\dagger e^{iM\boldsymbol{r}\cdot(\boldsymbol{b}_1+\boldsymbol{b}_2)/2} e^{i\pi M(M-1)/2} \quad [28]$$
$$= c_{\boldsymbol{r}+M\boldsymbol{a}_1}^\dagger e^{iM\boldsymbol{r}\cdot(\boldsymbol{b}_1+\boldsymbol{b}_2)/2} e^{i\pi M^2/2}. \quad [29]$$

Then for two-electron operators, in particular, we obtain:

$$T_{\boldsymbol{M}} c^\dagger_{\boldsymbol{r}} c^\dagger_{\boldsymbol{r}'} T^\dagger_{\boldsymbol{M}} = T_2^{M_2} T_1^{M_1} c^\dagger_{\boldsymbol{r}} c^\dagger_{\boldsymbol{r}'} T_1^{-M_1} T_2^{-M_2} \quad [30]$$

$$= e^{i\pi M_1^2} e^{iM_1(\boldsymbol{r}+\boldsymbol{r}')\cdot(\boldsymbol{b}_1+\boldsymbol{b}_2)/2} T_2^{M_2} c^\dagger_{\boldsymbol{r}+M_1\boldsymbol{a}_1} c^\dagger_{\boldsymbol{r}'+M_1\boldsymbol{a}_1} T_2^{-M_2} \quad [31]$$

$$= e^{i\pi(M_1^2+M_2^2)} e^{iM_1(\boldsymbol{r}+\boldsymbol{r}')\cdot(\boldsymbol{b}_1+\boldsymbol{b}_2)/2} e^{iM_2(\boldsymbol{r}+M_1\boldsymbol{a}_1)\cdot\boldsymbol{b}_2/2} e^{iM_2(\boldsymbol{r}'+M_1\boldsymbol{a}_1)\cdot\boldsymbol{b}_2/2} c^\dagger_{\boldsymbol{r}+\boldsymbol{M}} c^\dagger_{\boldsymbol{r}'+\boldsymbol{M}} \quad [32]$$

$$= e^{i\pi(M_1^2+M_2^2)} e^{iM_1(\boldsymbol{r}+\boldsymbol{r}')\cdot(\boldsymbol{b}_1+\boldsymbol{b}_2)/2} e^{iM_2\boldsymbol{r}\cdot\boldsymbol{b}_2/2} e^{iM_2\boldsymbol{r}'\cdot\boldsymbol{b}_2/2} c^\dagger_{\boldsymbol{r}+\boldsymbol{M}} c^\dagger_{\boldsymbol{r}'+\boldsymbol{M}}. \quad [33]$$

C. C_2 gauge for XC geometries. Fig. S1(b) displays the Hamiltonian hopping amplitudes that we employ for our calculations on the XC4 cylinder analyzed in the main text. In this gauge, the hoppings are real and imaginary. Moreover, the site-centered inversion symmetry is implemented as $C_2 c^\dagger_{\boldsymbol{r}} C_2^{-1} = c^\dagger_{C_2\boldsymbol{r}}$.

The lattice vector $\boldsymbol{a}_1$ points along the cylinder axis, while $2\boldsymbol{a}_2 - \boldsymbol{a}_1$ has length $\sqrt{3}$ and points along the circumference. For the XC4 infinite cylinder (1), the lattice and hoppings are repeated so that the cylinder has infinite length and circumference $2\sqrt{3}$. We also employ the hoppings of Fig. S1(b) in Sec. 4 of the *Supporting Information* below, where we calculate ground state correlation lengths on the XC4 and XC6 infinite cylinders at half filling.

2. Details of the U(1) Slave Rotor Theory and Mean Field Estimate of the Transition Point

We utilize the slave-rotor approach (2, 3), introducing a U(1) rotor variable $e^{i\theta}$ and its conjugate integer-valued "angular momentum" L on each site and writing the electron operator as

$$c^\dagger_{i,\sigma} = f^\dagger_{i,\sigma}\, e^{i\theta_i}, \quad [34]$$

where f_σ is a fermionic "spinon" that carries the spin index. The Hilbert space redundancy introduced by the rotors is removed by requiring that each site satisfy a constraint:

$$L_i = \sum_\sigma \left(f^\dagger_{i,\sigma} f_{i,\sigma} - 1/2 \right). \quad [35]$$

Note that the singly-filled site is represented by $L_i = 0$, while the doublon/empty sites are $L_i = \pm 1$. The Hubbard model can then be rewritten as:

$$H = -\sum_{\langle ij\rangle,\sigma} t_{ij} e^{i(\theta_i-\theta_j)} f^\dagger_{i,\sigma} f_{j,\sigma} + \text{h.c.} + \frac{U}{2}\sum_i L_i^2, \quad [36]$$

supplemented with the constraint Eq. (35) and the commutation relation $\left[e^{i\theta_i},\, L_j\right] = -\delta_{ij} e^{i\theta_i}$.

Of course, treating everything exactly is tantamount to solving the original problem, but we can make progress by adopting a mean field approach, satisfying the constraint *on average* and assigning self-consistent expectation values to $\langle f^\dagger_{i,\sigma} f_{j,\sigma}\rangle$ and $\langle e^{i(\theta_i-\theta_j)}\rangle$. This gives us the mean field Hamiltonian:

$$H_{MF} = H_f + H_\theta \quad [37]$$

$$H_f = -\sum_{\langle ij\rangle,\sigma} t_{ij} \langle e^{i(\theta_i-\theta_j)}\rangle f^\dagger_{i,\sigma} f_{j,\sigma} + \text{h.c.} \quad [38]$$

$$H_\theta = -\sum_{\langle ij\rangle,\sigma} t_{ij} e^{i(\theta_i-\theta_j)} \langle f^\dagger_{i,\sigma} f_{j,\sigma}\rangle + \text{h.c.} + \frac{U}{2}\sum_i L_i^2 \quad [39]$$

Note that the constraint Eq. (35) implies gauge invariance, $\theta_i \to \theta_i + \epsilon_i$, $f^\dagger_i \to f^\dagger_i e^{-i\epsilon_i}$ which is broken by the mean field Hamiltonian. This is remedied by incorporating a fluctuating gauge field a on the links, though we shall not do that here.

In the limit of small Hubbard U, we expect the rotor fields to condense, $\langle e^{i\theta_i}\rangle = \sqrt{Z_i} \leq 1$, so that the spinons f are identified with the electron up to this prefactor. This phase possesses off-diagonal long-range order; at the mean-field level, we assume $\langle e^{i(\theta_i-\theta_j)}\rangle \approx \langle e^{i\theta_i}\rangle\langle e^{-i\theta_j}\rangle$. Thus, the gap to the electron excitation is obtained from the dispersion $t_{ij}\sqrt{Z_i Z_j} f^\dagger_i f_j$, which explains why the single-electron gap on the IQH side (*i.e.*, the condensed rotor phase) *decreases* on increasing U.

On the other side of the phase diagram, when U is sufficiently large, we have $\langle e^{i\theta_i}\rangle = 0$ and thus the electron excitation is distinct from the spinon. To create an electron, one must both create a spinon $f^\dagger$ *and* increase the rotor angular momentum by $\Delta L = 1$. Thus, due to the interaction term, this gap tracks U for large U. Despite the absence of an expectation value $\langle e^{i\theta_i}\rangle = 0$ on this side, there is virtual tunneling of the rotor quanta, so that $\langle e^{i(\theta_i-\theta_j)}\rangle = \beta_{ij} \neq 0$ for neighboring sites (4). In fact, this expectation value is expected to scale as $|t|/U$, in the large U limit, implying that the dispersion of spinons in Eq. (39) is on the order of t^2/U, as is expected of magnetic excitations. On the other hand, if we assume that the expectation values β_{ij} are positive, consistent with the fact that the rotors see no net flux (while the electrons and spinons do), then we simply fill up the negative energy states of the band-structure shown in Fig. 1(c) of the main text, albeit with a smaller gap. This gives a non-zero value for the rotor hoppings, $\langle f^\dagger_{i,\sigma} f_{j,\sigma}\rangle = \alpha_{ij}$. We can find this expectation value from the ground state energy of the band Hamiltonian: writing $\alpha_{ij} = -t^*_{ij}\alpha/|t|$, we expect the average over the filled bands $\langle \epsilon^-(k)\rangle$ to determine $\alpha = \frac{|\langle \epsilon^-(k)\rangle|}{2z|t|}$, where $z = 6$ is the coordination number.

Now we can determine the transition, which amounts to analyzing the Bose-Hubbard model

$$H_B = -J\sum_{\langle ij\rangle} e^{i(\theta_i-\theta_j)} + \text{h.c.} + \frac{U}{2}\sum_i L_i^2. \qquad [40]$$

A simple mean field theory locates the transition at $U^* = 4zJ$. This is done by taking a simple variational ansatz for each site, $|\psi\rangle = |0\rangle + \frac{\psi}{2}\left(|+1\rangle + |-1\rangle\right)$ in the L basis, which has the property $\langle\psi|e^{i\theta}|\psi\rangle = \psi$. Then $\langle H_B\rangle = N\psi^2(-zJ + \frac{U}{4})$. The condensate occurs when the coefficient in parentheses first turns negative.

Substituting $J = 2|t|\alpha$, where the factor of 2 is for the two spins of f, we get:

$$U^* = 4|\langle\epsilon^-(k)\rangle| \approx 9.60813\,t \qquad [41]$$

where we averaged over the dispersion $\epsilon_-(k) = -2t\sqrt{\cos^2 k_1 + \cos^2 k_2 + \cos^2(k_1+k_2)}$. This mean field result is to be compared to, and is indeed relatively close to, the iDMRG-obtained transition that occurs at $U^*/t \approx 11-12$ (5, 6).

Additionally, we can argue directly that the gap to the charge-$2e$ Cooper pair excitations vanishes at the transition. To access them one has to include coupling to the internal gauge field a. If we focus on the transition and integrate out the fermionic spinons f, this gives a Chern-Simons term (7). Also, the rotor variables are at low energies and couple minimally to the gauge field. In the condensed phase of the rotors, we have the effective Lagrangian:

$$L = \frac{1}{4U}\left(\dot\theta + a_0\right)^2 - \frac{\rho_s}{2}\left(\nabla\theta + a\right)^2 + \frac{2}{4\pi}a\wedge da, \qquad [42]$$

where the superfluid density ρ_s goes to zero at the transition. The vortices of the rotor condensate are the Cooper pairs. The fact that they carry 2π flux of da implies, via the Chern-Simons term, a gauge charge of 2. This is screened by two rotor fields, giving the vortex a global $\mathrm{U}(1)_c$ charge of 2 and no spin. The energy of such vortices vanishes as we approach the transition.

3. Chirality of Edge States from K-Matrix Formalism

In this appendix, we provide a more thorough account of the charge response, spin response, and edge states of the IQH, CSL, and superconductor phases within the framework of Chern-Simons K-matrix formalism (8). We employ the following convention for the Chern-Simons Lagrangian:

$$\mathcal{L} = -\frac{K_{IJ}}{4\pi}\alpha_I\wedge d\alpha_J + \frac{t_J}{2\pi}A\wedge d\alpha_J + \frac{(t_s)_J}{2\pi}A_s\wedge d\alpha_J, \qquad [43]$$

or in components:

$$\mathcal{L} = -\frac{1}{4\pi}\alpha_I^\mu K_{IJ}\epsilon_{\mu\nu\lambda}\partial^\nu\alpha_J^\lambda + \frac{1}{2\pi}A^\mu t_J\epsilon_{\mu\nu\lambda}\partial^\nu\alpha_J^\lambda + \frac{1}{2\pi}(A_s)^\mu(t_s)_J\epsilon_{\mu\nu\lambda}\partial^\nu\alpha_J^\lambda. \qquad [44]$$

Here, α_I are dynamical U(1) gauge fields. On the other hand, A is a classical field that probes the charge response, while A_s probes the spin response. In order for this effective theory to describe a microscopic system of electrons, it must obey the "spin/charge relation", which is the following requirement on gauge-invariant *local* operators: those with odd electric charge must have fermionic statistics, while those with even electric charge have bosonic statistics (9). Mathematically, this condition reads (see Section 2.3 of Ref. (9)):

$$t_I \equiv K_{II} \pmod 2. \qquad [45]$$

This relation will be satisfied by the K-matrix theories for the IQH, CSL, and superconductor described below.

On a spatial manifold of genus g, the ground state degeneracy is given by $|\det K|^g$, provided $\det K \neq 0$. Furthermore, the edge chiral central charge is given by the "signature" of K, *i.e.*, c_- = the number of positive eigenvalues minus the number of negative eigenvalues (10). Provided the K-matrix is invertible, the Hall conductivity is (8)

$$\sigma_{xy} = \frac{e^2}{h}\sum_{IJ} t_I(K^{-1})_{IJ}t_J. \qquad [46]$$

and the spin quantum Hall conductivity (*i.e.*, spin current in response to a Zeeman gradient) is likewise

$$\sigma_{xy}^s = \frac{(\hbar/2)^2}{h}\sum_{IJ}(t_s)_I(K^{-1})_{IJ}(t_s)_J. \qquad [47]$$

Moreover, within the K-matrix formalism, each quasi-particle excitation can be labeled by a $\boldsymbol{\ell}$, where ℓ_I denotes its charge under each of the dynamical gauge fields. The current of this quasi-particle is then a source for the dynamical gauge fields and has statistical angle

$$\theta(\boldsymbol{\ell}) = \pi\sum_{IJ}\ell_I(K^{-1})_{IJ}\ell_J, \qquad [48]$$

electric charge

$$q(\boldsymbol{\ell}) = e\sum_{IJ}t_I(K^{-1})_{IJ}\ell_J, \qquad [49]$$

(where e is the electron charge) and S_z spin quantum number

$$q_s(\boldsymbol{\ell}) = \frac{\hbar}{2}\sum_{IJ}(t_s)_I(K^{-1})_{IJ}\ell_J. \qquad [50]$$

The spin-singlet integer quantum Hall phase (with total Chern number $C = 2$) is described by the data

$$K_{\text{IQH}} = \begin{pmatrix} 1 & 0 \\ 0 & 1 \end{pmatrix}, \qquad t_{\text{IQH}} = \begin{pmatrix} 1 \\ 1 \end{pmatrix}, \qquad t_{s,\text{IQH}} = \begin{pmatrix} 1 \\ -1 \end{pmatrix}. \qquad [51]$$

One immediately verifies that it satisfies the expected properties. It has a unique gapped ground state, as well as $c_- = 2$, $\sigma_{xy} = 2 \cdot e^2/h$, and $\sigma^s_{xy} = 2 \cdot (\hbar/2)^2/h$. Moreover, no quasi-particles are fractionalized: since $K_{\text{IQH}} = K^{-1}_{\text{IQH}} = 1_{2\times2}$, then $q(\boldsymbol{\ell}) = (\ell_1 + \ell_2)e$ and $q_s(\boldsymbol{\ell}) = (\ell_1 - \ell_2)\hbar/2$. Thus, in units of e and $\hbar/2$ respectively, $q(\boldsymbol{\ell})$ and $q_s(\boldsymbol{\ell})$ are both odd or both even.

The CSL phase is obtained by "gauging" the global charge U(1) of the IQH phase. Specifically, let us promote $A \to \alpha_3 + A$, where α_3 is a third dynamical U(1) gauge field and A is still a classical field. The resulting theory is described by the data

$$K_{\text{CSL}} = \begin{pmatrix} 1 & 0 & -1 \\ 0 & 1 & -1 \\ -1 & -1 & 0 \end{pmatrix}, \qquad t_{\text{CSL}} = \begin{pmatrix} 1 \\ 1 \\ 0 \end{pmatrix}, \qquad t_{s,\text{CSL}} = \begin{pmatrix} 1 \\ -1 \\ 0 \end{pmatrix}. \qquad [52]$$

The eigenvalues of K_{CSL} are $2, 1, -1$. Thus, the ground state degeneracy is 2^g and the chiral central charge is $c_- = 1$, as expected in the Kalmeyer-Laughlin CSL phase. Moreover, since

$$(K_{\text{CSL}})^{-1} = \frac{1}{2}\begin{pmatrix} 1 & -1 & -1 \\ -1 & 1 & -1 \\ -1 & -1 & -1 \end{pmatrix}, \qquad [53]$$

then this phase has the expected quantized Hall responses, $\sigma_{xy} = 0$ and $\sigma^s_{xy} = 2 \cdot (\hbar/2)^2/h$. Within this effective theory, the spin-up (spin-down) electron excitation is labeled by the first column $\boldsymbol{\ell}_{e\uparrow}$ (second column $\boldsymbol{\ell}_{e\downarrow}$) of K_{CSL}, with charges

$$q(\boldsymbol{\ell}_{e,\uparrow/\downarrow}) = e, \qquad q_s(\boldsymbol{\ell}_{e,\uparrow/\downarrow}) = \pm\hbar/2. \qquad [54]$$

On the other hand, there is a fractionalized charged semion excitation labeled by $\boldsymbol{\ell}_c = (0 \quad 0 \quad -1)^{\mathsf{T}}$, which carries $q(\boldsymbol{\ell}_c) = e, q_s(\boldsymbol{\ell}_c) = 0$, and a semionic statistical angle $\theta(\boldsymbol{\ell}_c) = -\pi/2$. The fractionalized spinful semion excitations correspond to $\boldsymbol{\ell}_{s,\uparrow/\downarrow} = \boldsymbol{\ell}_{e,\uparrow/\downarrow} - \boldsymbol{\ell}_c$, which indeed have $q(\boldsymbol{\ell}_s) = 0, q_s(\boldsymbol{\ell}_{s,\uparrow/\downarrow}) = \pm\hbar/2$, and a semionic statistical angle $\theta(\boldsymbol{\ell}_s) = +\pi/2$.

Now, let us turn to the anyon superconductor and the chirality of its edge modes. From the main text, recall that the main additional step was that the gauge field $a = \alpha_3$ now has a bIQH response $-\frac{2}{4\pi}ada$. With the sign convention of Eq. (43), adding this to K_{CSL} yields

$$K_{\text{SC}} = \begin{pmatrix} 1 & 0 & -1 \\ 0 & 1 & -1 \\ -1 & -1 & 2 \end{pmatrix}, \qquad t_{\text{SC}} = \begin{pmatrix} 1 \\ 1 \\ 0 \end{pmatrix}, \qquad t_{s,\text{SC}} = \begin{pmatrix} 1 \\ -1 \\ 0 \end{pmatrix}. \qquad [55]$$

We find $\det K_{\text{SC}} = 0$, which signals that the gauge fields are not independent and that the system (absent A) is gapless. Performing the change of variables

$$\begin{pmatrix} \alpha_1 \\ \alpha_2 \\ \alpha_3 \end{pmatrix} = \begin{pmatrix} \alpha'_1 + \alpha'_3 \\ \alpha'_2 + \alpha'_3 \\ \alpha'_3 \end{pmatrix} = W\begin{pmatrix} \alpha'_1 \\ \alpha'_2 \\ \alpha'_3 \end{pmatrix}, \qquad W = \begin{pmatrix} 1 & 0 & 1 \\ 0 & 1 & 1 \\ 0 & 0 & 1 \end{pmatrix}, \qquad [56]$$

we obtain the equivalent K-matrix theory

$$K'_{\text{SC}} = W^{\mathsf{T}}K_{\text{SC}}W = \begin{pmatrix} 1 & 0 & 0 \\ 0 & 1 & 0 \\ 0 & 0 & 0 \end{pmatrix}, \qquad t'_{\text{SC}} = W^{\mathsf{T}}t_{\text{SC}} = \begin{pmatrix} 1 \\ 1 \\ 2 \end{pmatrix}, \qquad t'_{s,\text{SC}} = W^{\mathsf{T}}t_{s,\text{SC}} = \begin{pmatrix} 1 \\ -1 \\ 0 \end{pmatrix}. \qquad [57]$$

This theory obeys the spin/charge relation Eq. (45), so that W is a valid transformation. Moreover, we see that α'_3 has decoupled from the other gauge fields and is governed by its Maxwell dynamics. With $A = 0$, this leads to a single bulk gapless mode corresponding to the superfluid Goldstone mode of the spontaneously-broken $\text{U}(1)_c$ symmetry. From the remaining fields, we see that there is no accompanying topological order and that the resulting phase is a chiral superfluid with edge chiral central charge $c_- = 2$.

Further insight into the topological properties of the superconductor is gained by replacing A with a 2+1D dynamical gauge field (11, 12). This gaps out the Goldstone mode of the superfluid and gives rise to one of Kitaev's 16-fold way $\mathbb{Z}_2$ topological orders (13). For instance, a topologically-trivial superfluid maps to the $m = 0$ member of the 16-fold way.* In fact, we only

*We use the symbol m instead of Kitaev's ν to avoid collision with the filling of the various quantum Hall phases.

expect one of the eight *even* members of the 16-fold way since we will only describe Abelian topological orders via the K-matrix. Making $\alpha_4' = A$ dynamical in Eq. (57) and discarding A_s, we obtain

$$K'_{\text{gauged SC}} = \begin{pmatrix} 1 & 0 & 0 & -1 \\ 0 & 1 & 0 & -1 \\ 0 & 0 & 0 & -2 \\ -1 & -1 & -2 & 0 \end{pmatrix}, \qquad [58]$$

which has precisely the same topological order as the gauged, weak-pairing $d+id$ superconductors (12). In particular, it corresponds to the member $m=4$ of Kitaev's classification (13), which has $c_- = m/2 = 2$. However, recall from the main text that our superconductor is far from the weak-pairing limit (evidenced by electron pairing above the insulators), so that its pairing symmetry is not directly linked to its edge chiral central charge (14).

4. Correlation Lengths at Half-Filling from Cylinder iDMRG

In this section, we provide the transfer matrix correlation length data for the ground states at half-filling, for a range of Hubbard interaction strengths U and various system sizes. We consider both the YC-L_y and XC-$2L_y$ cylinder geometries (1).

Let us first discuss the YC cylinder geometries. In the gauge-invariant language of Sec. 1 of the main text, in the YC case with even L_y, we take $T = T_2$ to be a purely circumferential translation with $T^{L_y} = 1$. When L_y is odd, we instead thread $\pi/2$ flux so that $T^{L_y} = i^{N_F}$, which means that T can have eigenvalue -1 in the charge-$2e$ ($N_F = 2$) sector. In both cases, the fluxes through the rings are such that the system respects particle-hole symmetry.

The XC case proceeds the same way except that we take our circumferential translation to be $T = (T_2)^2 T_1^\dagger$, which translates by a distance $\sqrt{3}a$ instead of a. For our Hamiltonian hopping amplitudes, we specifically choose those shown in Fig. S1(b).

The dominant correlation length ξ in a given charge sector of the transfer matrix is related to the dominant non-trivial transfer matrix eigenvalue λ by (15):

$$1/\xi_i = -\log|\lambda_i|. \qquad [59]$$

We report ξ in units of cylinder rings. For the YC cylinders, the distance between the cylinder rings is $a\sqrt{3}/2$, whereas it is $a/2$ in the XC case.

In Fig. S2, we display the maximum correlation lengths for the YC3, YC4 and YC6 cylinders resolved by the electric charge sector of the ground state transfer matrix, namely $Q \in \{0e, 1e, 2e\}$, where we maximize over spin and momentum quantum numbers for simplicity. We note that due to the pseudospin SU(2) symmetry, the charge-$0e$ and charge-$2e$ sectors manifestly have equal correlation lengths, with the exception of the regime $U/t \gtrsim 11$ where the spin-*triplet* charge-$0e$ branch (known to be low-energy from the ED and DMRG excitation calculations in the main text) have quantitatively larger correlation length, at least for the YC3 and YC4 systems. The main numerical feature we point out here, consistent with Ref. (6) (though differentiated here since we strictly study the pseudospin-symmetric configurations), is that the correlation length peak and the sharpness of the peak is in the charge-$0e/2e$ sectors is increasing with system size while the charge-$1e$ sector does not see a similar enhancement. The same features are seen in the XC4 vs. XC6 data, shown in Fig. S3.

5. Spin and Pseudospin SU(2) Formalism

A. Relation between spin and pseudospin generators. Here we present an alternative description, complementing the Majorana representation discussed in Sec. 1 of the main text, of how the presence of particle-hole symmetry and Hubbard interactions gives rise to the pseudospin SU$(2)_c$ symmetry (16). We begin with the familiar spin rotation generators:

$$S^+ = \sum_r c^\dagger_{r\uparrow} c_{r\downarrow}, \qquad S^- = \sum_r c^\dagger_{r\downarrow} c_{r\uparrow}, \qquad S^z = \frac{1}{2}\sum_r (n_{r\uparrow} - n_{r\downarrow}). \qquad [60]$$

Given a particle hole symmetry $\mathcal{P} c_{r\sigma} \mathcal{P}^{-1} = e^{i\alpha_r} c^\dagger_{r\sigma}$, which commutes with the Hamiltonian, let us define the following *half*-particle-hole transformation:

$$\bar{\mathcal{P}} c_{r\uparrow} \bar{\mathcal{P}}^{-1} = e^{i\alpha_r} c^\dagger_{r\uparrow}, \qquad \bar{\mathcal{P}} c_{r\downarrow} \bar{\mathcal{P}}^{-1} = c_{r\downarrow}. \qquad [61]$$

Though the half-PH leaves the spin-rotation-symmetric hopping Hamiltonian invariant, it is not a symmetry of the interaction since it flips the sign of the Hubbard U:

$$\bar{\mathcal{P}} \left(U \sum_r (n_{r\uparrow} - 1/2)(n_{r\downarrow} - 1/2) \right) \bar{\mathcal{P}}^{-1} = U \sum_r (1 - n_{r\uparrow} - 1/2)(n_{r\downarrow} - 1/2) = -U \sum_r (n_{r\uparrow} - 1/2)(n_{r\downarrow} - 1/2). \qquad [62]$$

Since H_{-U} also commutes with the spin generators, then $\bar{\mathcal{P}} S^\alpha \bar{\mathcal{P}}^{-1}$ are symmetries of H_{+U}. In particular, note that

$$\eta^+ \equiv \bar{\mathcal{P}} S^+ \bar{\mathcal{P}}^{-1} = \sum_r e^{-i\alpha_r} c_{r\uparrow} c_{r\downarrow}, \qquad [63]$$

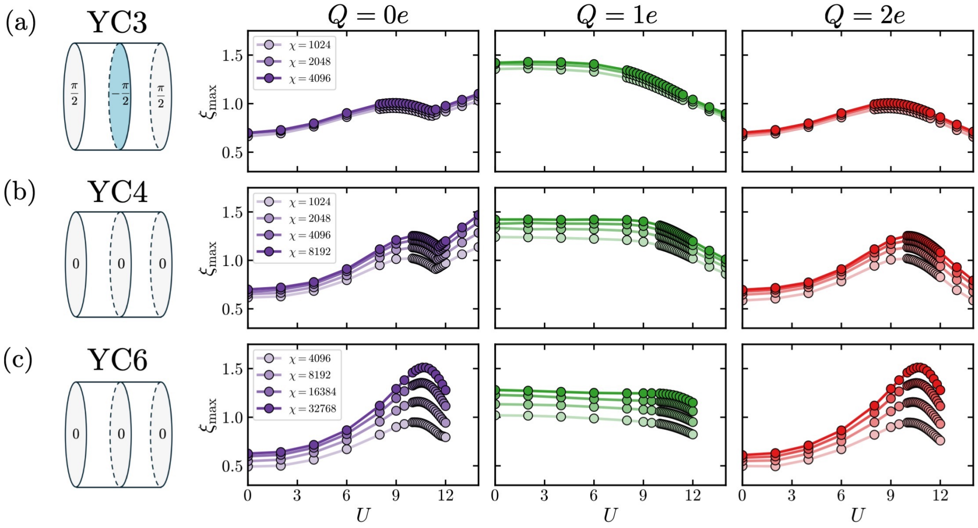

Fig. S2. (a) Maximum correlation length of the half-filled ground state transfer matrix on the infinite YC3 cylinder vs. Hubbard U in the charge sectors $Q \in \{0e, 1e, 2e\}$. The external flux is chosen so that the cylinder rings are pierced by fluxes $\pi/2, -\pi/2, \ldots$ which alternate between rings. Bond dimension, increasing with shade light-to-dark, indicated in legend. (b) Same for the YC4 infinite cylinder with zero flux through each ring. (c) Same for YC6 cylinder with zero flux through each ring.

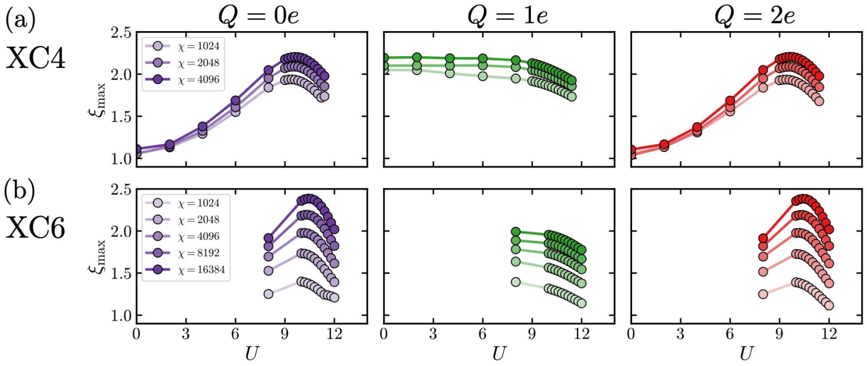

Fig. S3. (a) Maximum correlation length of the half-filled ground state transfer matrix on the infinite XC4 cylinder vs. Hubbard U in the charge sectors $Q \in \{0e, 1e, 2e\}$. Bond dimension, increasing light-to-dark with shade, indicated in legend. (b) Same for XC6 cylinder. For both the XC4 and XC6 cylinders, we make the choice of Hamiltonian hopping amplitudes specified in Sec. 1C of the *Supporting Information*.

while

$$\eta^z \equiv \bar{\mathcal{P}} S^z \bar{\mathcal{P}}^{-1} = \frac{1}{2}\sum_{\boldsymbol{r}}(1 - n_{\boldsymbol{r}\uparrow} - n_{\boldsymbol{r}\downarrow}) \qquad [64]$$

is related to the total charge density. While the resulting symmetries seem unnatural (in that η^z includes a minus sign relative to the charge density and η^+ *lowers* the total charge by $2e$), we will show later that this convention is the most natural for implementing spin and pseudospin rotations simultaneously as left and right actions, respectively, in a way that is consistent with $\bar{\mathcal{P}}$ relating the generators, as in Eqs. (6-8) of Ref. (17). Also, note that one can simply take $e^{i\alpha_{\boldsymbol{r}}} = 1$ when the hoppings are all imaginary, so that:

$$\mathcal{P} c_{\boldsymbol{r}\sigma} \mathcal{P}^{-1} = c^\dagger_{\boldsymbol{r}\sigma}. \qquad [65]$$

B. Fermionic matrix structure in $\mathrm{SU}(2)_s \times \mathrm{SU}(2)_c$ formalism. In order to get an analytic handle on how various $0e$ and $\pm 2e$ objects transform into one another under the pseudo SU(2) symmetry, it is useful to establish several identities. In particular, here we motivate defining the 2×2 fermion construction where spin (pseudospin) rotations act on the left (right). We know that the column vector of fermion annihilation operators transforms as a spin doublet: defining

$$C_{\boldsymbol{r}} = \begin{pmatrix} c_{\boldsymbol{r}\uparrow} \\ c_{\boldsymbol{r}\downarrow} \end{pmatrix}, \qquad [66]$$

then $[S_a, c_s] = -\frac{(\sigma_a)_{ss'}}{2} c_{s'}$, so that

$$[\boldsymbol{S}, C_{\boldsymbol{r}}] = -\frac{\boldsymbol{\sigma}}{2} C_{\boldsymbol{r}}, \qquad [\boldsymbol{S}, C^\dagger_{\boldsymbol{r}}] = C^\dagger_{\boldsymbol{r}} \frac{\boldsymbol{\sigma}}{2}. \qquad [67]$$

Exponentiating these relations, we get

$$e^{i\boldsymbol{\theta}\cdot\boldsymbol{S}} C_{\boldsymbol{r}} e^{-i\boldsymbol{\theta}\cdot\boldsymbol{S}} = e^{-i\boldsymbol{\theta}\cdot\boldsymbol{\sigma}/2} C_{\boldsymbol{r}}. \qquad [68]$$

Now it's easy to obtain the analogous relation for the pseudospin SU(2) generators via the mapping $\boldsymbol{\eta} = \bar{\mathcal{P}} \boldsymbol{S} \bar{\mathcal{P}}^\dagger$. We simply conjugate both sides by $\bar{\mathcal{P}}$ and use the fact that $\bar{\mathcal{P}} C_{\boldsymbol{r}} \bar{\mathcal{P}}^\dagger = \begin{pmatrix} c^\dagger_{\boldsymbol{r}\uparrow} \\ c_{\boldsymbol{r}\downarrow} \end{pmatrix}$. For the infinitesimal relations, we obtain $[\boldsymbol{\eta}, \begin{pmatrix} c^\dagger_{\boldsymbol{r}\uparrow} \\ c_{\boldsymbol{r}\downarrow} \end{pmatrix}] = -\frac{\boldsymbol{\sigma}}{2} \begin{pmatrix} c^\dagger_{\boldsymbol{r}\uparrow} \\ c_{\boldsymbol{r}\downarrow} \end{pmatrix}$, or by taking the Hermitian conjugate,

$$\left[\boldsymbol{\eta}, \begin{pmatrix} c_{\boldsymbol{r}\uparrow} & c^\dagger_{\boldsymbol{r}\downarrow} \end{pmatrix}\right] = \begin{pmatrix} c_{\boldsymbol{r}\uparrow} & c^\dagger_{\boldsymbol{r}\downarrow} \end{pmatrix} \frac{\boldsymbol{\tau}}{2}. \qquad [69]$$

(we have relabeled the Pauli matrices as $\boldsymbol{\tau}$ when they act to the right as pseudospin rotations). Exponentiating gives

$$e^{i\boldsymbol{\theta}\cdot\boldsymbol{\eta}} \begin{pmatrix} c_{\boldsymbol{r}\uparrow} & c^\dagger_{\boldsymbol{r}\downarrow} \end{pmatrix} e^{-i\boldsymbol{\theta}\cdot\boldsymbol{\eta}} = \begin{pmatrix} c_{\boldsymbol{r}\uparrow} & c^\dagger_{\boldsymbol{r}\downarrow} \end{pmatrix} e^{i\boldsymbol{\theta}\cdot\boldsymbol{\tau}/2}. \qquad [70]$$

Thus $\begin{pmatrix} c_{\boldsymbol{r}\uparrow} & c^\dagger_{\boldsymbol{r}\downarrow} \end{pmatrix}$ should be a row of the 2×2 fermion matrix. It turns out that completing this matrix with $-c^\dagger_{\boldsymbol{r}\uparrow}$ as the bottom right element makes both columns (rows) transform as spin (charge) doublets. Namely,

$$\Psi_{\boldsymbol{r}} = \begin{pmatrix} c_{\boldsymbol{r}\uparrow} & c^\dagger_{\boldsymbol{r}\downarrow} \\ c_{\boldsymbol{r}\downarrow} & -c^\dagger_{\boldsymbol{r}\uparrow} \end{pmatrix} = \begin{pmatrix} C_{\boldsymbol{r}} & i\sigma_y C^*_{\boldsymbol{r}} \end{pmatrix} \qquad [71]$$

satisfies

$$e^{i\boldsymbol{\theta}\cdot\boldsymbol{S}} \Psi_{\boldsymbol{r}} e^{-i\boldsymbol{\theta}\cdot\boldsymbol{S}} = e^{-i\boldsymbol{\theta}\cdot\boldsymbol{\sigma}/2} \Psi_{\boldsymbol{r}} \qquad [72]$$

and, on account of the relation $\bar{\mathcal{P}} C_{\boldsymbol{r}} \bar{\mathcal{P}}^\dagger = C^\dagger_{\boldsymbol{r}}$, also satisfies

$$e^{i\boldsymbol{\theta}\cdot\boldsymbol{\eta}} \Psi_{\boldsymbol{r}} e^{-i\boldsymbol{\theta}\cdot\boldsymbol{\eta}} = \Psi_{\boldsymbol{r}} e^{i\boldsymbol{\theta}\cdot\boldsymbol{\tau}/2}. \qquad [73]$$

We also remark that the spin and charge generators can be neatly written in terms of this fermionic matrix. First:

$$\boldsymbol{S}_{\boldsymbol{r}} = \frac{1}{4}\mathrm{tr}\left(\Psi^\dagger_{\boldsymbol{r}} \boldsymbol{\sigma} \Psi_{\boldsymbol{r}}\right), \qquad [74]$$

which naturally transforms as a vector under spin rotations. Using the half-PH change of basis, we directly obtain

$$\boldsymbol{\eta}_{\boldsymbol{r}} = \bar{\mathcal{P}} \boldsymbol{S} \bar{\mathcal{P}}^\dagger = \frac{1}{4}\mathrm{tr}\left(\Psi_{\boldsymbol{r}} \boldsymbol{\tau} \Psi^\dagger_{\boldsymbol{r}}\right), \qquad [75]$$

where we have once again trivially rewritten the charge Pauli matrices as $\boldsymbol{\tau}$.

Finally, we note a useful implementation of the Hermitian conjugate:

$$\Psi^\dagger_{\boldsymbol{r}} = \begin{pmatrix} c^\dagger_{\boldsymbol{r}\uparrow} & c^\dagger_{\boldsymbol{r}\downarrow} \\ c_{\boldsymbol{r}\downarrow} & -c_{\boldsymbol{r}\uparrow} \end{pmatrix} = -\sigma_y \begin{pmatrix} c_{\boldsymbol{r}\uparrow} & c_{\boldsymbol{r}\downarrow} \\ c^\dagger_{\boldsymbol{r}\downarrow} & -c^\dagger_{\boldsymbol{r}\uparrow} \end{pmatrix} \sigma_y = -\tau_y \Psi^{\mathsf{T}}_{\boldsymbol{r}} \sigma_y. \qquad [76]$$

This can be useful when contracting multiple $\Psi_{\boldsymbol{r}}$ under a trace, but one has to be careful about extra minus signs from fermion anti-commutation.

C. Hofstadter-Hubbard Hamiltonian. Suppose we are given a hopping model $h = -\sum_{\boldsymbol{r},\boldsymbol{r}'} t_{\boldsymbol{r}\boldsymbol{r}'} c^\dagger_{\boldsymbol{r}} c_{\boldsymbol{r}'}$ where $\boldsymbol{r}, \boldsymbol{r}'$ each run over all the sites in the triangular lattice. As usual, Hermiticity requires that

$$t_{\boldsymbol{r}\boldsymbol{r}'} = t^*_{\boldsymbol{r}'\boldsymbol{r}}. \tag{77}$$

If we further suppose that the *hoppings are all imaginary*, then

$$t_{\boldsymbol{r}\boldsymbol{r}'} = -t_{\boldsymbol{r}'\boldsymbol{r}}. \tag{78}$$

In particular, this means the Hamiltonian has the particle-hole symmetry $\mathcal{P} c_{\boldsymbol{r}} \mathcal{P}^{-1} = c^\dagger_{\boldsymbol{r}}$, like our $\Phi_\triangle = \pi/2$ Hofstadter model on the triangular lattice. In the general case, we claim that the hopping Hamiltonian can be written as

$$h = -\sum_{\boldsymbol{r} \leftarrow \boldsymbol{r}'} t_{\boldsymbol{r}\boldsymbol{r}'} \operatorname{tr}\left(\Psi^\dagger_{\boldsymbol{r}} \Psi_{\boldsymbol{r}'}\right), \tag{79}$$

where the sum is over *oriented* pairs of sites, which we denote by $\boldsymbol{r} \leftarrow \boldsymbol{r}'$, whose orientation we fix so that $t_{\boldsymbol{r}\boldsymbol{r}'} = +it$ (with $t > 0$) which gives

$$h = -it \sum_{\boldsymbol{r} \leftarrow \boldsymbol{r}'} \operatorname{tr}\left(\Psi^\dagger_{\boldsymbol{r}} \Psi_{\boldsymbol{r}'}\right). \tag{80}$$

The Hamiltonian is already Hermitian in this form. To see this, we explicitly compute its Hermitian conjugate:

$$h^\dagger = -\sum_{\boldsymbol{r} \leftarrow \boldsymbol{r}'} t^*_{\boldsymbol{r}\boldsymbol{r}'} \operatorname{tr}\left(\Psi^\dagger_{\boldsymbol{r}'} \Psi_{\boldsymbol{r}}\right) = +\sum_{\boldsymbol{r} \leftarrow \boldsymbol{r}'} t_{\boldsymbol{r}\boldsymbol{r}'} \operatorname{tr}\left(\left(-\tau_y \Psi^\intercal_{\boldsymbol{r}'} \sigma_y\right)\left(-\sigma_y (\Psi^\dagger_{\boldsymbol{r}})^\intercal \tau_y\right)\right), \tag{81}$$

where we used Eq. (76). After the Pauli matrices and minus signs cancel, we anti-commute the two operators (they live on different sites) and confirm that

$$h^\dagger = -\sum_{\boldsymbol{r} \leftarrow \boldsymbol{r}'} t_{\boldsymbol{r}\boldsymbol{r}'} \operatorname{tr}\left((\Psi^\dagger_{\boldsymbol{r}})^\intercal \Psi^\intercal_{\boldsymbol{r}'}\right) = -\sum_{\boldsymbol{r} \leftarrow \boldsymbol{r}'} t_{\boldsymbol{r}\boldsymbol{r}'} \operatorname{tr}\left(\Psi^\dagger_{\boldsymbol{r}} \Psi_{\boldsymbol{r}'}\right) = h. \tag{82}$$

We note that it is manifestly spin- and pseudospin-rotation-invariant due to the trace. To verify that h is indeed the familiar hopping Hamiltonian, we explicitly expand out the expression:

$$h = -\sum_{\boldsymbol{r} \leftarrow \boldsymbol{r}'} t_{\boldsymbol{r}\boldsymbol{r}'} \operatorname{tr}\left(\begin{pmatrix} c^\dagger_{\boldsymbol{r}\uparrow} & c^\dagger_{\boldsymbol{r}\downarrow} \\ c_{\boldsymbol{r}\downarrow} & -c_{\boldsymbol{r}\uparrow} \end{pmatrix}\begin{pmatrix} c_{\boldsymbol{r}'\uparrow} & c^\dagger_{\boldsymbol{r}'\downarrow} \\ c_{\boldsymbol{r}'\downarrow} & -c^\dagger_{\boldsymbol{r}'\uparrow} \end{pmatrix}\right) \tag{83}$$

$$= -\sum_{\boldsymbol{r} \leftarrow \boldsymbol{r}'} (t_{\boldsymbol{r}\boldsymbol{r}'} c^\dagger_{\boldsymbol{r}s} c_{\boldsymbol{r}'s} + t_{\boldsymbol{r}\boldsymbol{r}'} c_{\boldsymbol{r}s} c^\dagger_{\boldsymbol{r}'s}) \tag{84}$$

$$= -\sum_{\boldsymbol{r} \leftarrow \boldsymbol{r}'} (t_{\boldsymbol{r}\boldsymbol{r}'} c^\dagger_{\boldsymbol{r}s} c_{\boldsymbol{r}'s} + t^*_{\boldsymbol{r}\boldsymbol{r}'} c^\dagger_{\boldsymbol{r}'s} c_{\boldsymbol{r}s}) \tag{85}$$

$$= -\sum_{\boldsymbol{r} \leftarrow \boldsymbol{r}'} (t_{\boldsymbol{r}\boldsymbol{r}'} c^\dagger_{\boldsymbol{r}s} c_{\boldsymbol{r}'s} + h.c.), \tag{86}$$

where we again used the fact that $\boldsymbol{r} \neq \boldsymbol{r}'$, and for the last equality we crucially used the imaginary condition, Eq. (78).

As for the Hubbard interaction, we reference Ref. (17):

$$H_U = \frac{2U}{3} \sum_{\boldsymbol{r}} \left(\eta^2_{\boldsymbol{r}} - \frac{3}{8}\right), \tag{87}$$

which is clearly also invariant under both spin (because $[\boldsymbol{\eta}, \boldsymbol{S}] = 0$) and pseudospin rotations.

6. SU(2) Slave-Rotor Theory of IQH-CSL transition

The presence of both $\mathrm{SU}(2)_s$ and pseudospin symmetries $\mathrm{SU}(2)_c$ enables an SU(2) slave-rotor analysis of the IQH, CSL, and their critical point. The construction was introduced by Hermele in Ref. (17) in the context of Hubbard models on bipartite lattices, but applies in the present context with almost no modification. The main difference is that since the triangular lattice has no natural sublattice, we instead work in the Imaginary C6 gauge (with all imaginary hoppings), where the particle-hole acts as in Eq. (65) above. This means we don't need to keep track of a sublattice-dependent sign, *i.e.*, Hermele's $\epsilon_{A/B} = \pm 1$.

A. Physical Hilbert space from parton constraint. We begin by decomposing the electron operators into spinons and rotors:

$$\Psi_{\boldsymbol{r}} = F_{\boldsymbol{r}} Z_{\boldsymbol{r}}, \qquad F_{\boldsymbol{r}} = \begin{pmatrix} f_{\boldsymbol{r}\uparrow} & f^\dagger_{\boldsymbol{r}\downarrow} \\ f_{\boldsymbol{r}\downarrow} & -f^\dagger_{\boldsymbol{r}\uparrow} \end{pmatrix}, \qquad Z_{\boldsymbol{r}} = \begin{pmatrix} z_{\boldsymbol{r}1} & z_{\boldsymbol{r}2} \\ -z^\dagger_{\boldsymbol{r}2} & z^\dagger_{\boldsymbol{r}1} \end{pmatrix}. \qquad [88]$$

The spinon operators are arranged like the electron operators, with spin acting by SU(2) rotations from the left, whereas gauge rotations act from the right. The rotor $Z_{\boldsymbol{r}}$ is an SU(2) matrix of operators (meaning $z^\dagger_{\boldsymbol{r}1} z_{\boldsymbol{r}1} + z^\dagger_{\boldsymbol{r}2} z_{\boldsymbol{r}2} = 1$), with gauge rotations acting from the left and pseudospin rotations acting from the right. The enlarged slave-rotor Hilbert space at each site is a product of that of the spin-1/2 spinons and that of the SU(2) rotor. Since the slave-rotor Hilbert space is much larger than the physical one, we have to specify which of its states are physical. Following Ref. (17), one projects to the subspace of gauge singlets, $\boldsymbol{J}_G(\boldsymbol{r}) = 0$, where:

$$\boldsymbol{J}_G(\boldsymbol{r}) = \frac{1}{4}\mathrm{tr}\left(F_{\boldsymbol{r}} \boldsymbol{\mu} F^\dagger_{\boldsymbol{r}}\right) + \frac{1}{4}\mathrm{tr}\left(Z^\dagger_{\boldsymbol{r}} \boldsymbol{\mu} Z_{\boldsymbol{r}}\right), \qquad [89]$$

so that

$$e^{i\boldsymbol{\alpha}\cdot\boldsymbol{J}_G(\boldsymbol{r})} Z_{\boldsymbol{r}} e^{-i\boldsymbol{\alpha}\cdot\boldsymbol{J}_G(\boldsymbol{r})} = e^{-i\boldsymbol{\alpha}\cdot\boldsymbol{\mu}/2} Z_{\boldsymbol{r}}, \qquad [90]$$

$$e^{i\boldsymbol{\alpha}\cdot\boldsymbol{J}_G(\boldsymbol{r})} F_{\boldsymbol{r}} e^{-i\boldsymbol{\alpha}\cdot\boldsymbol{J}_G(\boldsymbol{r})} = F_{\boldsymbol{r}} e^{i\boldsymbol{\alpha}\cdot\boldsymbol{\mu}/2}. \qquad [91]$$

We have denoted the Pauli matrices acting in the gauge space by $\boldsymbol{\mu}$, reserving $\boldsymbol{\sigma}$ for the spin space and $\boldsymbol{\tau}$ for the pseudospin space, as in Sec. B above.

Note that the F-Z Hamiltonian—obtained by writing the Hamiltonian in terms of partons—is a gauge-singlet operator. It therefore acts within the $\boldsymbol{J}_G(\boldsymbol{r}) = 0$ subspace and has the same matrix elements as the electronic Hamiltonian. To construct this subspace, which is four-dimensional at each site, we start with the slave-particle vacuum:

$$|0\rangle_{\mathrm{sp}} = |0\rangle_f \otimes |0\rangle_{\mathrm{rot}}, \qquad [92]$$

where $|0\rangle_f$ is the spinon vacuum and $|0\rangle_{\mathrm{rot}} = |\ell_C = 0, \ell_G = 0, m_C = 0, m_G = 0\rangle$ is the unique rotationally-invariant state in the rotor Hilbert space (C stands for "charge" pseudospin and G stands for gauge); recall from Ref. (17) that only $\ell_G = \ell_C$ states are allowed to begin with. In particular, $\boldsymbol{J}_G(\boldsymbol{r})|0\rangle_{\mathrm{rot}} = 0$ by definition. Then it can be shown that while $|0\rangle_{\mathrm{sp}}$ is not a physical state, $f^\dagger_{\boldsymbol{r}\uparrow}|0\rangle_{\mathrm{sp}}$ is physical, *i.e.*, it is a gauge-singlet. Similarly, $f^\dagger_{\boldsymbol{r}\downarrow}|0\rangle_{\mathrm{sp}}$ is a physical state. We can then construct the fully-filled and empty states by applying electronic operators:

$$c^\dagger_{\boldsymbol{r}\uparrow} f^\dagger_{\boldsymbol{r}\downarrow}|0\rangle_{\mathrm{sp}} = (f^\dagger_{\boldsymbol{r}\uparrow} z^\dagger_{\boldsymbol{r}1} - f_{\boldsymbol{r}\downarrow} z_{\boldsymbol{r}2}) f^\dagger_{\boldsymbol{r}\downarrow}|0\rangle_{\mathrm{sp}} = (z^\dagger_{\boldsymbol{r}1} f^\dagger_{\boldsymbol{r}\uparrow} f^\dagger_{\boldsymbol{r}\downarrow} - z_{\boldsymbol{r}2})|0\rangle_{\mathrm{sp}}, \qquad [93]$$

and

$$c_{\boldsymbol{r}\uparrow} f^\dagger_{\boldsymbol{r}\uparrow}|0\rangle_{\mathrm{sp}} = (f_{\boldsymbol{r}\uparrow} z_{\boldsymbol{r}1} - f^\dagger_{\boldsymbol{r}\downarrow} z^\dagger_{\boldsymbol{r}2}) f^\dagger_{\boldsymbol{r}\uparrow}|0\rangle_{\mathrm{sp}} = (z_{\boldsymbol{r}1} + z^\dagger_{\boldsymbol{r}2} f^\dagger_{\boldsymbol{r}\uparrow} f^\dagger_{\boldsymbol{r}\downarrow})|0\rangle_{\mathrm{sp}}. \qquad [94]$$

Since the electron operators are invariant under gauge transformations, then both of these states are gauge singlets. We have therefore generated the full four-dimensional physical on-site Hilbert space.

B. Functional integral representation. Following the path integral construction outlined in Ref. (17), the electronic Hofstadter-Hubbard Hamiltonian can now be written in the slave-rotor representation:

$$H_{\mathrm{HH}} = -\sum_{\boldsymbol{r},\boldsymbol{r}'} \mathrm{tr}\left[(F_{\boldsymbol{r}} Z_{\boldsymbol{r}})^\dagger (F_{\boldsymbol{r}'} Z_{\boldsymbol{r}'})\right] + \frac{2U}{3} \sum_{\boldsymbol{r}} \eta^2_{\boldsymbol{r}}, \qquad [95]$$

which must be accompanied by the aforementioned "gauge-singlet" constraint

$$\boldsymbol{J}_{\boldsymbol{G}}(\boldsymbol{r}) = 0. \qquad [96]$$

Working at zero temperature, we introduce imaginary time $\tau \in (-\infty, +\infty)$ and write the functional integral:

$$\mathcal{Z} = \int \mathcal{D}Z \mathcal{D}F \mathcal{D}\boldsymbol{a}_\tau e^{-S}, \qquad S = S_Z + S_F + S_t, \qquad [97]$$

where

$$S_Z = \frac{3}{4U} \sum_{\boldsymbol{r}} \int d\tau\ \mathrm{tr}\left[Z^\dagger_{\boldsymbol{r}} \left(\overleftarrow{\partial}_\tau - \frac{i\boldsymbol{a}_\tau \cdot \boldsymbol{\mu}}{2}\right)\left(\partial_\tau + \frac{i\boldsymbol{a}_\tau \cdot \boldsymbol{\mu}}{2}\right) Z_{\boldsymbol{r}}\right], \qquad [98]$$

$$S_F = \frac{1}{2} \sum_{\boldsymbol{r}} \int d\tau\ \mathrm{tr}\left[F_{\boldsymbol{r}} \left(\partial_\tau + \frac{i\boldsymbol{a}_\tau \cdot \boldsymbol{\mu}}{2}\right) F^\dagger_{\boldsymbol{r}}\right], \qquad [99]$$

$$S_t = -it \int d\tau \sum_{\boldsymbol{r}\leftarrow\boldsymbol{r}'} \mathrm{tr}\left[(F_{\boldsymbol{r}} Z_{\boldsymbol{r}})^\dagger (F_{\boldsymbol{r}'} Z_{\boldsymbol{r}'})\right]. \qquad [100]$$

Here and below, $\overleftarrow{\partial}$ denotes differentiation acting to the left, and the gauge field $\boldsymbol{a}_\tau$ has been introduced to enforce the on-site gauge constraint (Eq. (96)).

So far everything is exact and therefore intractable, so we pass to a mean-field approximation. The first step is to replace the constraint $Z_{\boldsymbol{r}}(\tau) \in \mathrm{SU}(2)$ with an equivalent Lagrange multiplier term:

$$S_\lambda = i \int d\tau \sum_{\boldsymbol{r}} \lambda_{\boldsymbol{r}}(\tau) \left[\frac{1}{2}\mathrm{tr}(Z_{\boldsymbol{r}}^\dagger Z_{\boldsymbol{r}}) - 1\right]. \qquad [101]$$

The next step is to decouple the hopping term into separate spinon and rotor terms. This is done by introducing a complex Hubbard–Stratonovich field via the identity (17, 18):

$$e^{\epsilon \alpha_{\boldsymbol{r}\boldsymbol{r}'} \beta_{\boldsymbol{r}\boldsymbol{r}'}} = \frac{\epsilon}{\pi} \int d\eta_{\boldsymbol{r}\boldsymbol{r}'} d\eta^*_{\boldsymbol{r}\boldsymbol{r}'}\ e^{-\epsilon(|\eta_{\boldsymbol{r}\boldsymbol{r}'}|^2 - \eta_{\boldsymbol{r}\boldsymbol{r}'}\alpha_{\boldsymbol{r}\boldsymbol{r}'} - \eta^*_{\boldsymbol{r}\boldsymbol{r}'}\beta_{\boldsymbol{r}\boldsymbol{r}'})}.$$

Since we have fixed the bond orientation for the hopping in Eq. (100) above, and since $S_t = t\int d\tau \sum_{\boldsymbol{r}\leftarrow\boldsymbol{r}'}(-Z_{\boldsymbol{r}'}Z_{\boldsymbol{r}}^\dagger)_{ab}(iF_{\boldsymbol{r}}^\dagger F_{\boldsymbol{r}'})_{ba}$, then we may assign $\epsilon = t\Delta\tau > 0$ and for each a, b assign $\alpha^{ab}_{\boldsymbol{r}\boldsymbol{r}'} = -(Z_{\boldsymbol{r}'}Z_{\boldsymbol{r}}^\dagger)_{ab}$ and $\beta_{\boldsymbol{r}\boldsymbol{r}'} = (iF_{\boldsymbol{r}}^\dagger F_{\boldsymbol{r}'})_{ba}$, which yields:

$$S_\eta = t \int d\tau \sum_{\boldsymbol{r}\leftarrow\boldsymbol{r}'} \mathrm{tr}\left[(\eta_{\boldsymbol{r}\boldsymbol{r}'})^\dagger \eta_{\boldsymbol{r}\boldsymbol{r}'}\right],$$

$$S_{tZ} = -t \int d\tau \sum_{\boldsymbol{r}\leftarrow\boldsymbol{r}'} \mathrm{tr}\left[Z_{\boldsymbol{r}}^\dagger \eta_{\boldsymbol{r}\boldsymbol{r}'} Z_{\boldsymbol{r}'}\right],$$

$$S_{tF} = it \int d\tau \sum_{\boldsymbol{r}\leftarrow\boldsymbol{r}'} \mathrm{tr}\left[F_{\boldsymbol{r}'}(\eta_{\boldsymbol{r}\boldsymbol{r}'})^\dagger F_{\boldsymbol{r}}^\dagger\right].$$

One obtains a real free energy at the saddle point provided that $\eta_{\boldsymbol{r}\boldsymbol{r}'} = \chi_{\boldsymbol{r}\boldsymbol{r}'} U_{\boldsymbol{r}\boldsymbol{r}'}$ is a real number times an SU(2) matrix, as can be shown using the identity Eq. (76) above. In particular, two of the saddle point equations read $(\eta_{\boldsymbol{r}\boldsymbol{r}'})^\dagger = \langle Z_{\boldsymbol{r}'} Z_{\boldsymbol{r}}^\dagger\rangle$ and $\eta_{\boldsymbol{r}\boldsymbol{r}'} = (t_{\boldsymbol{r}\boldsymbol{r}'}/|t_{\boldsymbol{r}\boldsymbol{r}'}|)\langle F_{\boldsymbol{r}}^\dagger F_{\boldsymbol{r}'}\rangle = i\langle F_{\boldsymbol{r}}^\dagger F_{\boldsymbol{r}'}\rangle$. We take an ansatz where $\langle F_{\boldsymbol{r}}^\dagger F_{\boldsymbol{r}'}\rangle$ is proportional to $\langle \Psi_{\boldsymbol{r}}^\dagger \Psi_{\boldsymbol{r}'}\rangle$ in the $U = 0$ IQH phase, producing a solution $\eta^*_{\boldsymbol{r}\boldsymbol{r}'}$ (at $\boldsymbol{a}_\tau = 0$).

Fluctuations about this solution are parameterized by an SU(2) gauge-field on the links $\boldsymbol{r} \leftarrow \boldsymbol{r}'$ and by a fluctuating $\boldsymbol{a}_\tau$ component. Integrating out the spinons while retaining these gauge field degrees of freedom, then in the continuum limit we obtain at leading order in the gauge fields (19):

$$S_{\mathrm{CS}} = \frac{i}{4\pi}\int d^3x\ \epsilon^{\mu\nu\rho}\mathrm{tr}\left(a_\mu \partial_\nu a_\rho + \frac{2}{3}a_\mu a_\nu a_\rho\right),$$

which is an SU(2) Chern-Simons term at level 1 (20).

C. Low-energy $\mathrm{SU}(2)_1$ Higgs-Chern-Simons theory. To describe the IQH-CSL transition, we pass to a continuum description of the rotor bosons. We begin with the mean-field action

$$S^Z_{\mathrm{MF}} = -\sum_{\tau,\boldsymbol{r}}\left[\mathrm{tr}Z^\dagger_{x+\epsilon z}Z_x + h.c.\right] - \sum_\tau \sum_{\langle \boldsymbol{r},\boldsymbol{r}'\rangle}\left[\mathrm{tr}Z^\dagger_{\boldsymbol{r},\tau}Z_{\boldsymbol{r}',\tau} + h.c.\right] + \frac{r + r_{c0}}{2}\sum_{\tau,\boldsymbol{r}}\left[\mathrm{tr}Z_x^\dagger Z_x\right], \qquad [102]$$

where r_{c0} is chosen so that the lowest boson mode goes gapless as $r \to 0^+$. Following Ref. (17), we take a spacetime lattice of triangular lattice sites separated by ϵ increments in imaginary time. For $r > 0$, diagonalizing the above spacetime-translation-invariant Hamiltonian gives a unique low-energy mode at $\boldsymbol{q} = 0$, consistent with the rotors experiencing no net flux. We write $\Theta(x) \sim Z_{\boldsymbol{r},t}$ with x now a continuous spacetime coordinate, which yields:

$$\mathcal{L}^\Theta_0 = \frac{1}{2}\mathrm{tr}(\Theta^\dagger \overleftarrow{\partial}_\nu \partial_\nu \Theta) + \frac{r}{2}\mathrm{tr}(\Theta^\dagger\Theta), \qquad [103]$$

where $\overleftarrow{\partial}$ denotes differentiation to the left. Note that demanding invariance under global SU(2) gauge transformations and pseudospin rotations precludes a linear time-derivative term $\sim \mathrm{tr}(\Theta^\dagger \partial_\tau \Theta)$. This entirely fixes the form at $\mathcal{L}^\Theta_0$ quadratic order. Now we add the gauge field fluctuations by promoting the derivative to the covariant derivative

$$\partial_\nu \to \partial_\nu + \frac{i\boldsymbol{a}_\nu \cdot \boldsymbol{\mu}}{2}, \qquad [104]$$

giving

$$\mathcal{L}^\Theta_{\mathrm{kin}} = \frac{1}{2}\mathrm{tr}\left[\Theta^\dagger\left(\overleftarrow{\partial}_\mu - \frac{ia^j_\mu \sigma^i}{2}\right)\left(\overrightarrow{\partial}_\mu + \frac{ia^j_\mu\sigma^i}{2}\right)\Theta\right], \qquad [105]$$

where $\boldsymbol{\mu}$ is again the Pauli matrix vector in the gauge space. Reintroducing the $\mathrm{SU}(2)_1$ Chern-Simons term from integrating out the spinons, and the symmetry-allowed quartic term, the full SU(2) Higgs-Chern-Simons theory is:

$$\mathcal{L}_{\mathrm{eff}} = \mathcal{L}_{\mathrm{CS}} + \mathcal{L}^\Theta_{\mathrm{kin}} + \frac{1}{2}\mathrm{tr}(\Theta^\dagger\Theta) + \frac{\lambda}{4}\left[\mathrm{tr}(\Theta^\dagger\Theta)\right]^2 + \ldots \qquad [106]$$

D. Conserved current. As spin excitations are gapped across the IQH-CSL transition, and neither the spin nor pseudospin symmetries are broken, then the closing of the charge gap is associated with gapless bosonic modes that transform as spin-singlet pseudospin-triplets. We now compute the conserved Noether current associated with the pseudospin rotation $\mathrm{SU}(2)_c$ symmetry. The important Lagrangian term for the current is the coupling $\mathcal{L}^{\Theta}_{\text{kin}}$ between the low-energy bosonic field Θ and the SU(2) gauge field in Eq. (105) above. Under the pseudospin SU(2) rotation

$$\Theta' = \Theta e^{i\boldsymbol{\alpha}\cdot\boldsymbol{\tau}/2} \approx \Theta + \Theta\frac{i\boldsymbol{\alpha}\cdot\boldsymbol{\tau}}{2}, \tag{107}$$

this term transforms to

$$(\mathcal{L}^{\Theta}_{\text{kin}})' = \frac{1}{2}\text{tr}\left[\left(\Theta^\dagger - \frac{i\boldsymbol{\alpha}\cdot\boldsymbol{\tau}}{2}\Theta^\dagger\right)\left(\overleftarrow{\partial}_\mu - \frac{ia^j_\mu\sigma^i}{2}\right)\left(\vec{\partial}_\mu + \frac{ia^j_\mu\sigma^i}{2}\right)\left(\Theta + \Theta\frac{i\boldsymbol{\alpha}\cdot\boldsymbol{\tau}}{2}\right)\right] \tag{108}$$

$$= \mathcal{L}^{\Theta}_{\text{kin}} + \frac{1}{2}\text{tr}\left[\left(-\frac{i\boldsymbol{\alpha}\cdot\boldsymbol{\tau}}{2}\Theta^\dagger\right)\left(\overleftarrow{\partial}_\mu - \frac{ia^j_\mu\sigma^i}{2}\right)\left(\vec{\partial}_\mu + \frac{ia^j_\mu\sigma^i}{2}\right)\Theta\right] \tag{109}$$

$$+ \frac{1}{2}\text{tr}\left[\Theta^\dagger\left(\overleftarrow{\partial}_\mu - \frac{ia^j_\mu\sigma^i}{2}\right)\left(\vec{\partial}_\mu + \frac{ia^j_\mu\sigma^i}{2}\right)\left(\Theta\frac{i\boldsymbol{\alpha}\cdot\boldsymbol{\tau}}{2}\right)\right]. \tag{110}$$

When α is constant, then $(\mathcal{L}^{\Theta}_{\text{kin}})' = \mathcal{L}^{\Theta}_{\text{kin}}$ to all orders. When it varies in spacetime, on the other hand

$$(\mathcal{L}^{\Theta}_{\text{kin}})' - \mathcal{L}^{\Theta}_{\text{kin}} = \frac{1}{2}\text{tr}\left[\left(-\frac{i\boldsymbol{\alpha}\cdot\boldsymbol{\tau}}{2}\Theta^\dagger\right)\left(\overleftarrow{\partial}_\mu - \frac{ia^j_\mu\sigma^i}{2}\right)\left(\vec{\partial}_\mu + \frac{ia^j_\mu\sigma^i}{2}\right)\Theta\right] \tag{111}$$

$$+ \frac{1}{2}\text{tr}\left[\Theta^\dagger\left(\overleftarrow{\partial}_\mu - \frac{ia^j_\mu\sigma^i}{2}\right)\left(\vec{\partial}_\mu + \frac{ia^j_\mu\sigma^i}{2}\right)\left(\Theta\frac{i\boldsymbol{\alpha}\cdot\boldsymbol{\tau}}{2}\right)\right]. \tag{112}$$

After expansion and simplification, the two terms above reduce to:

$$\frac{1}{2}\text{tr}\left[\Theta^\dagger\left(\overleftarrow{\partial}_\mu - \frac{ia^j_\mu\sigma^i}{2}\right)\left(\vec{\partial}_\mu + \frac{ia^j_\mu\sigma^i}{2}\right)\left(\Theta\frac{i\boldsymbol{\alpha}\cdot\boldsymbol{\tau}}{2}\right)\right] = \frac{1}{2}\text{tr}\left[\Theta^\dagger\left(\overleftarrow{\partial}_\mu - \frac{ia^j_\mu\sigma^i}{2}\right)\Theta\frac{i\partial_\mu\boldsymbol{\alpha}\cdot\boldsymbol{\tau}}{2}\right] \tag{113}$$

$$+ \frac{1}{2}\text{tr}\left[\Theta^\dagger\left(\overleftarrow{\partial}_\mu - \frac{ia^j_\mu\sigma^i}{2}\right)\left(\vec{\partial}_\mu + \frac{ia^j_\mu\sigma^i}{2}\right)\Theta \times \frac{i\boldsymbol{\alpha}\cdot\boldsymbol{\tau}}{2}\right], \tag{114}$$

and likewise

$$\frac{1}{2}\text{tr}\left[\left(-\frac{i\boldsymbol{\alpha}\cdot\boldsymbol{\tau}}{2}\Theta^\dagger\right)\left(\overleftarrow{\partial}_\mu - \frac{ia^j_\mu\sigma^i}{2}\right)\left(\vec{\partial}_\mu + \frac{ia^j_\mu\sigma^i}{2}\right)\Theta\right] = -\frac{1}{2}\text{tr}\left[\frac{i\partial_\mu\boldsymbol{\alpha}\cdot\boldsymbol{\tau}}{2}\Theta^\dagger\left(\vec{\partial}_\mu + \frac{ia^j_\mu\sigma^i}{2}\right)\Theta\right] \tag{115}$$

$$- \frac{1}{2}\text{tr}\left[\frac{i\boldsymbol{\alpha}\cdot\boldsymbol{\tau}}{2} \times \Theta^\dagger\left(\overleftarrow{\partial}_\mu - \frac{ia^j_\mu\sigma^i}{2}\right)\left(\vec{\partial}_\mu + \frac{ia^j_\mu\sigma^i}{2}\right)\Theta\right]. \tag{116}$$

By cyclicity of the trace, what remains after cancellation is:

$$(\mathcal{L}^{\Theta}_{\text{kin}})' - \mathcal{L}^{\Theta}_{\text{kin}} = \partial_\mu\alpha^j \cdot \frac{1}{2}\text{tr}\left[\Theta^\dagger\left(\overleftarrow{\partial}_\mu - \frac{ia^j_\mu\sigma^i}{2}\right)\Theta\frac{i\tau^j}{2} - \frac{i\tau^j}{2}\Theta^\dagger\left(\vec{\partial}_\mu + \frac{ia^j_\mu\sigma^i}{2}\right)\Theta\right], \tag{117}$$

so that the conserved current is

$$\boldsymbol{\mathcal{J}}_\mu \propto \text{tr}\left[\frac{\boldsymbol{\tau}}{2}\frac{\Theta^\dagger\left(\vec{\partial}_\mu + \frac{ia^j_\mu\sigma^i}{2}\right)\Theta - \Theta^\dagger\left(\overleftarrow{\partial}_\mu - \frac{ia^j_\mu\sigma^i}{2}\right)\Theta}{2i}\right] = \text{tr}\left[\frac{\boldsymbol{\tau}}{2}\frac{\Theta^\dagger\vec{D}_\mu\Theta - \Theta^\dagger\overleftarrow{D}_\mu\Theta}{2i}\right]. \tag{118}$$

Because of the pseudospin Pauli vector $\boldsymbol{\tau}/2$, this bosonic object transforms as a vector under global pseudospin rotations $\Theta \to \Theta e^{i\boldsymbol{\theta}\cdot\boldsymbol{\tau}/2}$. Indeed, its τ^z component is like the density of low-energy Cooper pairs, while $\tau^\pm$ serve as Cooper pair annihilation/creation operators. Moreover, it is gauge-invariant since $D_\mu\Theta$ is gauge-covariant. Finally, since it is the conserved current, then at the critical point all components will have protected scaling dimension 2. Altogether, this SU(2) formulation confirms the physical conclusions made in Ref. (21), by working here in a formalism manifestly invariant under the pseudospin symmetry.

7. Electron Pairing from Small-U Diagrammatic Expansion

A. Band basis in the doubly-folded Brillouin zone. Let's work in the Imaginary C6 gauge, which has four sublattice sites $\mathcal{S} = \{A, B, C, D\}$, arranged as in Fig. S1(a). While T_1 and T_2 anti-commute in the odd-fermion-parity sector, $(T_1)^2$ and $(T_2)^2$ commute. Thus, given displacements $\boldsymbol{d} \in \bar{\mathcal{L}} \equiv 2\mathcal{L}$ (*i.e.* in the A sublattice), then the operators $T_{\boldsymbol{d}}$ have unambiguous action and *form a group.* In fact, in the Imaginary C_6 gauge with single-site translations defined as in Eq. (5), these two-site translation operators are *pure coordinate shift*, as shown in Eq. (6) above. Therefore we can readily apply Bloch's theorem with a four-site unit cell. Let $\overline{\mathrm{BZ}}$ denote the "doubly-folded" Brillouin zone corresponding to $\bar{\mathcal{L}}$. The Fourier-transformed electron operators are

$$c^\dagger_{\boldsymbol{k},\gamma} = \frac{1}{\sqrt{|\bar{\mathcal{L}}|}} \sum_{\boldsymbol{d}\in\bar{\mathcal{L}}} e^{i\boldsymbol{k}\cdot(\boldsymbol{d}+\boldsymbol{\gamma})} T_{\boldsymbol{d}} c^\dagger_{\boldsymbol{0}+\boldsymbol{\gamma}} T^\dagger_{\boldsymbol{d}} = \frac{1}{\sqrt{|\bar{\mathcal{L}}|}} \sum_{\boldsymbol{d}\in\bar{\mathcal{L}}} e^{i\boldsymbol{k}\cdot(\boldsymbol{d}+\boldsymbol{\gamma})} c^\dagger_{\boldsymbol{d}+\boldsymbol{\gamma}}, \qquad [119]$$

where we used the fact that $T_{\boldsymbol{d}}$ is pure coordinate shift for $\boldsymbol{d} \in \bar{\mathcal{L}}$.

Now let's Fourier transform the hopping Hamiltonian, namely

$$h = -\sum_{\boldsymbol{r},\boldsymbol{r}'} t_{\boldsymbol{r}\boldsymbol{r}'} c^\dagger_{\boldsymbol{r}} c_{\boldsymbol{r}'} = -\sum_{\gamma,\gamma'} \sum_{\boldsymbol{d},\boldsymbol{d}'\in\bar{\mathcal{L}}} t_{\boldsymbol{d}+\boldsymbol{\gamma},\boldsymbol{d}'+\boldsymbol{\gamma}'} c^\dagger_{\boldsymbol{d}+\boldsymbol{\gamma}} c_{\boldsymbol{d}'+\boldsymbol{\gamma}'}, \qquad [120]$$

where γ, γ' run over the sublattices $\mathcal{S}$ and

$$t_{\boldsymbol{d}+\boldsymbol{\gamma},\boldsymbol{d}'+\boldsymbol{\gamma}'} = t_{\gamma,\gamma'}(\boldsymbol{d}-\boldsymbol{d}') \qquad [121]$$

is manifestly translation-invariant on the scale of $2\mathcal{L}$. Inverting the electron Fourier transform Eq. (119), we obtain

$$c^\dagger_{\boldsymbol{d}+\boldsymbol{\gamma}} = \sum_{\boldsymbol{k}\in\overline{\mathrm{BZ}}} e^{-i\boldsymbol{k}\cdot(\boldsymbol{d}+\boldsymbol{\gamma})} c^\dagger_{\boldsymbol{k},\gamma}, \qquad [122]$$

and substituting it in gives

$$h = -\frac{1}{|\bar{\mathcal{L}}|} \sum_{\gamma,\gamma'} \sum_{\boldsymbol{d},\boldsymbol{d}'\in\bar{\mathcal{L}}} t_{\boldsymbol{d}+\boldsymbol{\gamma},\boldsymbol{d}'+\boldsymbol{\gamma}'} \left(\sum_{\boldsymbol{k}\in\overline{\mathrm{BZ}}} e^{-i\boldsymbol{k}\cdot(\boldsymbol{d}+\boldsymbol{\gamma})} c^\dagger_{\boldsymbol{k},\gamma} \right) \left(\sum_{\boldsymbol{k}'\in\overline{\mathrm{BZ}}} e^{i\boldsymbol{k}'\cdot(\boldsymbol{d}'+\boldsymbol{\gamma}')} c_{\boldsymbol{k}',\gamma'} \right) \qquad [123]$$

$$= \sum_{\boldsymbol{k},\boldsymbol{k}'\in\overline{\mathrm{BZ}}} \sum_{\gamma,\gamma'} \left[\sum_{\boldsymbol{\delta}\in\bar{\mathcal{L}}} -e^{-i\boldsymbol{k}\cdot\boldsymbol{\delta}} e^{-i\boldsymbol{k}\cdot\boldsymbol{\gamma}} t_{\gamma,\gamma'}(\boldsymbol{\delta}) e^{i\boldsymbol{k}'\cdot\boldsymbol{\gamma}'} \right] \left(\frac{1}{|\bar{\mathcal{L}}|} \sum_{\boldsymbol{d}'\in\bar{\mathcal{L}}} e^{i(\boldsymbol{k}'-\boldsymbol{k})\cdot\boldsymbol{d}'} \right) c^\dagger_{\boldsymbol{k},\gamma} c_{\boldsymbol{k}',\gamma'} \qquad [124]$$

$$= \sum_{\boldsymbol{k}\in\overline{\mathrm{BZ}}} \sum_{\gamma,\gamma'} c^\dagger_{\boldsymbol{k},\gamma} h_{\gamma,\gamma'}(\boldsymbol{k}) c_{\boldsymbol{k},\gamma'}, \qquad [125]$$

where

$$h_{\gamma,\gamma'}(\boldsymbol{k}) = e^{-i\boldsymbol{k}\cdot\boldsymbol{\gamma}} \left(-\sum_{\boldsymbol{\delta}\in\bar{\mathcal{L}}} e^{-i\boldsymbol{k}\cdot\boldsymbol{\delta}} t_{\gamma,\gamma'}(\boldsymbol{\delta}) \right) e^{i\boldsymbol{k}'\cdot\boldsymbol{\gamma}'}. \qquad [126]$$

Using $t_{\gamma',\gamma}(-\boldsymbol{\delta})^* = t_{\gamma,\gamma'}(\boldsymbol{\delta})$, one can show that $h_{\gamma,\gamma'}(\boldsymbol{k})$ is a Hermitian matrix in the sublattice. We plot the higher of the two resulting bands in Fig. 1(c) of the main text. Note that the lower and upper bands are each two-fold degenerate, a consequence of our having folded the lattice-scale Brillouin zone not once but twice (in order to accommodate the 2×2-site unit cell and C_6 rotations).

Let us denote the magnetic reciprocal lattice vectors of the magnetic lattice by

$$\bar{\mathcal{L}}^{-1} \in \mathrm{span}\,\{\boldsymbol{b}_1/2, \boldsymbol{b}_2/2\} \qquad [127]$$

(where the "span" is over integer linear combinations), defined equivalently as those vectors for which $\boldsymbol{\delta} \in \bar{\mathcal{L}} \implies \boldsymbol{G}\cdot\boldsymbol{\delta} \in 2\pi\mathbb{Z}$. Under shifts by such vectors, the Hamiltonian transforms as

$$h_{\gamma,\gamma'}(\boldsymbol{k}+\boldsymbol{G}) = e^{-i\boldsymbol{G}\cdot\boldsymbol{\gamma}} h_{\gamma,\gamma'}(\boldsymbol{k}) e^{i\boldsymbol{G}\cdot\boldsymbol{\gamma}'}. \qquad [128]$$

As 4×4 matrices in the sublattice degree of freedom, $h(\boldsymbol{k}+\boldsymbol{G})$ is therefore unitarily-related to $h(\boldsymbol{k})$ and has the same spectrum. We obtain eigenstates by diagonalization:

$$h(\boldsymbol{k})u(\boldsymbol{k}) = u(\boldsymbol{k})E(\boldsymbol{k}), \qquad [129]$$

where $E(\boldsymbol{k})$ is a diagonal matrix of energies, while $u_{\gamma,n}(\boldsymbol{k})$ is a unitary matrix whose columns are labeled by band n and whose rows are labeled by sublattice γ. Correspondingly, the Bloch fermion creation operators are

$$f^\dagger_n(\boldsymbol{k}) = \sum_\gamma c^\dagger_{\boldsymbol{k},\gamma} u_{\gamma,n}(\boldsymbol{k}), \qquad [130]$$

which is easily shown to diagonalize the hopping Hamiltonian and satisfy the usual fermion anti-commutation relations.

B. Perturbative demonstration of odd-angular momentum spin-singlet pairing. We rewrite the model in momentum space

$$\mathcal{H} = \sum_{12} c_2^\dagger h_{2,1} c_1 + \frac{1}{2N} \sum_{1234} c_4^\dagger c_3^\dagger \Gamma_{43,21} c_2 c_1, \qquad [131]$$

with N the number of unit cells, and $i = (k_i, o_i, \sigma_i)$ a generalized index gathering the single particle momentum k_i belonging to the Brillouin zone (BZ), the sublattice index o_i, and the spin σ_i. Due to momentum and spin conservation, and because our model only features a Hubbard interaction, the parameters in this Hamiltonian can be simplified as

$$\begin{aligned} h_{2,1} &= \delta_{k_1,k_2} \delta_{\sigma_1,\sigma_2} h_{o_2,o_1}(k_1), \\ \Gamma_{43,21} &= U \delta_{s_4,s_1,\uparrow} \delta_{s_3,s_2,\downarrow} \delta_{o_4,o_3,o_2,o_1} \delta_{k_1+k_2,k_3+k_4}, \end{aligned} \qquad [132]$$

Renormalization of the two-body scattering vertex is described, to second order in many-body perturbation theory, by the five diagrams (22):

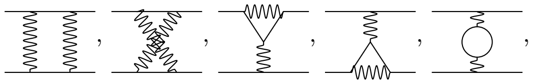 [133]

where curvy and straight lines respectively denote interaction events and single-particle propagators. The evaluation of these diagrams is detailed in App. D of Ref. (23), producing a renormalized interaction vertex $\Gamma^{\text{eff}} = \Gamma + \delta\Gamma$, with

$$\delta\Gamma_{43,21} = -\frac{1}{N} \sum_{abcd} \chi^-_{dc,ba} \Gamma_{43,ba} \Gamma_{dc,21} + \chi^+_{dc,ba} [\Gamma_{4c,2a}\Gamma_{d3,b1} + \Gamma_{c3,2a}\Gamma_{d4,b1} + \Gamma_{4c,a1}\Gamma_{d3,b2} - N_f \Gamma_{4c,a1}\Gamma_{d3,2b}], \qquad [134]$$

where the terms are ordered as their corresponding diagrams in Eq. (133) where $N_f = 2$ is the number of spin species. The sum over the generalized indices (a, b, c, d) runs over momenta, orbital and spin indices. The single-particle propagators are characterized by the Bloch eigenvectors $\Psi^{o_i}_{k_i,n}$ with n a band index, which are the same for both spin species. Finally, we have respectively denoted as χ^- and χ^+ the particle-particle and particle-hole susceptibilities, whose explicit form is

$$\chi^{\epsilon=\pm}_{dc,ba} = \delta^{k_d}_{k_a} \delta^{k_c}_{k_b} \sum_{n,n'=\pm} \Psi^{o_d\,*}_{k_d,n} \Psi^{o_c\,*}_{k_c,n'} \Psi^{o_b}_{k_b,n'} \Psi^{o_a}_{k_a,n} \frac{f_\beta(\epsilon \xi_{k_a,n}) - f_\beta(\xi_{k_b,n'})}{\xi_{k_a,n} - \epsilon \xi_{k_b,n'}},$$

where $f_\beta(x) = 1/[1 + e^{\beta x}]$ is the Fermi-Dirac distribution, and $\xi_i = \varepsilon_i - \mu$ measures energies with respect to the chemical potential μ.

We are interested in the correction to the Cooper interaction $\delta\Gamma_{(k',\uparrow,o_4)(-k',\downarrow,o_3),(-k,\downarrow,o_2)(k,\uparrow,o_1)}$ at zero temperature when the chemical potential is in the middle of the gap. Several simplifications arise:

- First, the contributions of bubble and of the upper and lower wine glass diagrams vanish. This can be straightforwardly checked using the spin constraints on the bare vertex Γ.
- Second, we can see that χ^- only involves intra-band processes that are most relevant in presence of a Fermi surface, *i.e.*, at finite doping which is not the focus of this pairing calculation. This simply comes from $f_\beta(\xi < 0) = 1$ and $f_\beta(\xi > 0) = 0$.
- This only leaves the crossed diagram.

Focusing on the Cooper channel, we evaluate this crossed diagram and project onto the given bands (in the Imaginary C6 gauge, there are two bands per valley above half-filling) to get

$$\delta\Gamma_{(k',\uparrow,n_4)(-k',\downarrow,n_3),(-k,\downarrow,n_2)(k,\uparrow,n_1)} = -U^2 \sum_{o,o'} \left(\Psi^{n_4}_{k',o} \Psi^{n_3}_{-k',o'}\right)^* \tilde{\chi}_{o',o}(k'+k) \left(\Psi^{n_2}_{-k,o} \Psi^{n_1}_{k,o'}\right), \qquad [135]$$

where the orbital susceptibilities are defined by

$$\tilde{\chi}_{o',o}(q) = \frac{1}{N} \sum_p \chi^+_{(p+q,o')(p,o),(p,o')(p+q,o)}. \qquad [136]$$

Note that in spite of the overall sign in front of the sum, this interaction is mostly repulsive because the susceptibilities defined above are negative in the density-density channel where $o = o'$.

If we focus on the effective scattering coefficient between electrons occupying the lowest single-particle states of the model, then we can replace k and k' by κ/κ'. Then there are only three orbital susceptibilities to compute $\tilde{\chi}(0, \kappa, \kappa')$ and four Bloch states to obtain in order to evaluate all the different Cooper scattering coefficients. This forms a matrix that we can diagonalize to identify the most attractive (if any). We have computed all of those terms, and have found a single attractive channel that we write in the basis where band **0** has C_3 eigenvalues ω at κ/κ', bands **1** and **3** have zero angular momentum at those points, and band **2** has C_3 eigenvalue of ω^*. All of these single-electron angular momenta were computed using the conventions for gauge and C_6 described in App. B above.

We focus on the conduction bands **2** and **3**. The pairing channel we find is a spin-singlet and carries C_3 angular momentum (one electron of the pair in each band) in our choice of gauge and for our choice of rotation symmetry C_3. Examining at the pair closely, it is antisymmetric in the band index and in the valley index (hence fully antisymmetric under particle exchange, as required). Altogether, we can write the pair down as:

$$\hat{\Delta} = c_{\kappa',\uparrow,\omega^*} c_{\kappa,\downarrow,1} + c_{\kappa,\uparrow,1} c_{\kappa',\downarrow,\omega^*} - c_{\kappa',\uparrow,1} c_{\kappa,\downarrow,\omega^*} - c_{\kappa,\uparrow,\omega^*} c_{\kappa',\downarrow,1}. \quad [137]$$

The valley antisymmetry, corresponding to eigenvalue -1 under C_2, makes explicit the *odd site-centered angular momentum nature of the pair.* Recall the discussion in the main text that $C_3 \to (e^{2\pi i/3})^{N_F} C_3$ may be redefined, thus modifying the C_3 eigenvalue, while preserving the identity $(C_3)^3 = 1$. Therefore, to prevent confusion, in the main text we have replaced the C_3 eigenvalue labels 1 and ω^* with an index $n \in \{1, 2\}$ which likewise labels the degenerate bands at each of κ and κ'.

References

1. A Szasz, J Motruk, MP Zaletel, JE Moore, Chiral spin liquid phase of the triangular lattice hubbard model: A density matrix renormalization group study. *Phys. Rev. X* **10**, 021042 (2020).
2. S Florens, A Georges, Quantum impurity solvers using a slave rotor representation. *Phys. Rev. B* **66**, 165111 (2002).
3. S Florens, A Georges, Slave-rotor mean-field theories of strongly correlated systems and the mott transition in finite dimensions. *Phys. Rev. B* **70**, 035114 (2004).
4. Z Song, UFP Seifert, ZX Luo, L Balents, Mott insulators in moiré transition metal dichalcogenides at fractional fillings: Slave-rotor mean-field theory. *Phys. Rev. B* **108**, 155109 (2023).
5. C Kuhlenkamp, W Kadow, Amc Imamoğlu, M Knap, Chiral pseudospin liquids in moiré heterostructures. *Phys. Rev. X* **14**, 021013 (2024).
6. S Divic, T Soejima, V Crépel, MP Zaletel, A Millis, Chiral Spin Liquid and Quantum Phase Transition in the Triangular Lattice Hofstadter-Hubbard Model. *arXiv e-prints* p. arXiv:2406.15348 (2024).
7. XL Qi, TL Hughes, SC Zhang, Topological field theory of time-reversal invariant insulators. *Phys. Rev. B* **78**, 195424 (2008).
8. XG Wen, A Zee, Classification of abelian quantum hall states and matrix formulation of topological fluids. *Phys. Rev. B* **46**, 2290–2301 (1992).
9. N Seiberg, E Witten, Gapped boundary phases of topological insulators via weak coupling. *Prog. Theor. Exp. Phys.* **2016**, 12C101 (2016).
10. YM Lu, A Vishwanath, Classification and properties of symmetry-enriched topological phases: Chern-simons approach with applications to Z_2 spin liquids. *Phys. Rev. B* **93**, 155121 (2016).
11. T Hansson, V Oganesyan, S Sondhi, Superconductors are topologically ordered. *Annals Phys.* **313**, 497–538 (2004).
12. S Moroz, A Prem, V Gurarie, L Radzihovsky, Topological order, symmetry, and hall response of two-dimensional spin-singlet superconductors. *Phys. Rev. B* **95**, 014508 (2017).
13. A Kitaev, Anyons in an exactly solved model and beyond. *Annals Phys.* **321**, 2–111 (2006) January Special Issue.
14. N Read, D Green, Paired states of fermions in two dimensions with breaking of parity and time-reversal symmetries and the fractional quantum hall effect. *Phys. Rev. B* **61**, 10267–10297 (2000).
15. V Zauner, et al., Transfer matrices and excitations with matrix product states. *New J. Phys.* **17**, 053002 (2015).
16. M Shiroishi, H Ujino, M Wadati, (4) symmetry of the transfer matrix for the one-dimensional hubbard model. *J. Phys. A: Math. Gen.* **31**, 2341 (1998).
17. M Hermele, Su(2) gauge theory of the hubbard model and application to the honeycomb lattice. *Phys. Rev. B* **76**, 035125 (2007).
18. SS Lee, PA Lee, U(1) gauge theory of the hubbard model: Spin liquid states and possible application to $\kappa-(\mathrm{BEDT-TTF})_2\mathrm{cu}_2(\mathrm{CN})_3$. *Phys. Rev. Lett.* **95**, 036403 (2005).
19. XG Wen, Non-abelian statistics in the fractional quantum hall states. *Phys. Rev. Lett.* **66**, 802–805 (1991).
20. E Witten, Quantum field theory and the Jones polynomial. *Commun. Math. Phys.* **121**, 351 – 399 (1989).
21. JY Lee, C Wang, MP Zaletel, A Vishwanath, YC He, Emergent multi-flavor qed$_3$ at the plateau transition between fractional chern insulators: Applications to graphene heterostructures. *Phys. Rev. X* **8**, 031015 (2018).
22. W Kohn, JM Luttinger, New mechanism for superconductivity. *Phys. Rev. Lett.* **15**, 524–526 (1965).
23. V Crépel, T Cea, L Fu, F Guinea, Unconventional superconductivity due to interband polarization. *Phys. Rev. B* **105**, 094506 (2022).
